# The Role of Reactive Oxygen Species in Antibiotic-Induced Cell Death in *Burkholderia cepacia* Complex Bacteria

**DOI:** 10.1371/journal.pone.0159837

**Published:** 2016-07-20

**Authors:** Heleen Van Acker, Jan Gielis, Marloes Acke, Freya Cools, Paul Cos, Tom Coenye

**Affiliations:** 1 Laboratory of Pharmaceutical Microbiology, Ghent University, Ghent, Belgium; 2 Department of Thoracic Surgery, Antwerp Surgical Training and Anatomy Research Centre (ASTARC), Antwerp University, Antwerp, Belgium; 3 Laboratory for Microbiology, Parasitology and Hygiene, University of Antwerp, Antwerp, Belgium; The Scripps Research Institute and Sorrento Therapeutics, Inc., UNITED STATES

## Abstract

It was recently proposed that bactericidal antibiotics, besides through specific drug-target interactions, kill bacteria by a common mechanism involving the production of reactive oxygen species (ROS). However, this mechanism involving the production of hydroxyl radicals has become the subject of a lot of debate. Since the contribution of ROS to antibiotic mediated killing most likely depends on the conditions, differences in experimental procedures are expected to be at the basis of the conflicting results. In the present study different methods (ROS specific stainings, gene-expression analyses, electron paramagnetic resonance, genetic and phenotypic experiments, detection of protein carbonylation and DNA oxidation) to measure the production of ROS upon antibiotic treatment in *Burkholderia cepacia* complex (*Bcc*) bacteria were compared. Different classes of antibiotics (tobramycin, ciprofloxacin, meropenem) were included, and both planktonic and biofilm cultures were studied. Our results indicate that some of the methods investigated were not sensitive enough to measure antibiotic induced production of ROS, including the spectrophotometric detection of protein carbonylation. Secondly, other methods were found to be useful only in specific conditions. For example, an increase in the expression of OxyR was measured in *Burkholderia cenocepacia* K56-2 after treatment with ciprofloxacin or meropenem (both in biofilms and planktonic cultures) but not after treatment with tobramycin. In addition results vary with the experimental conditions and the species tested. Nevertheless our data strongly suggest that ROS contribute to antibiotic mediated killing in *Bcc* species and that enhancing ROS production or interfering with the protection against ROS may form a novel strategy to improve antibiotic treatment.

## Introduction

A natural side effect of aerobic respiration is the production of reactive oxygen species (ROS) [1]. These ROS are generated via successive single-electron reductions and can damage DNA, proteins and lipids, ultimately leading to cell death. To protect themselves against the deleterious effects of ROS, aerobic bacteria are equipped with enzymes (catalases and superoxide dismutases) that can detoxify ROS and regulatory mechanisms (SoxRS, OxyRS, and SOS regulons) to counter the damage [2]. Interestingly, in 2007 Kohanski et al. identified a common mechanism involving the production of hydroxyl radicals by which all bactericidal antibiotics could induce cell death [3]. Currently, a mechanism is proposed in which bacterial membrane disturbance triggers envelope stress and subsequent perturbation of the Arc regulatory system accelerates respiration [4]. Hyperactivation of the electron transport chain induces the formation of superoxide and hydrogen peroxide which damage iron-sulphur clusters, thereby releasing ferrous iron. This iron can then react with hydrogen peroxide in the Fenton reaction and generate hydroxyl radicals which can directly damage DNA, lipids and proteins or oxidize the deoxynucleotide pool and indirectly damage DNA. However, this theory has recently become the subject of much debate [5–8]. A major point of criticism is the use of hydroxyphenyl fluorescein as a stain to demonstrate ROS production [9], although various studies have used other direct (chemiluminescence, electron paramagnetic resonance (EPR)) or indirect methods (quantification of protein carbonylation or expression of proteins involved in antioxidant strategies) to confirm production of ROS [10–12]. Moreover, it was found that protection against ROS has a positive effect on bacterial cell survival not only after treatment with oxidizing agents but also after treatment with antibiotics [2, 13]. Most studies investigating the contribution of ROS in antibiotic-mediated killing have focused on planktonic cultures, but cells in a biofilm may respond differently. For example, for *Pseudomonas aeruginosa* biofilms higher bactericidal concentrations were needed to induce ROS production compared to planktonic cultures [14], and it has been shown that ciprofloxacin only induces oxidative stress in planktonic *Proteus mirabilis* cells but not in biofilms [15]. ROS production most likely contributes to antibiotic-mediated killing, but the extent depends on the specific conditions [16, 17]. Hence, differences in experimental procedures could be at the basis of the conflicting results reported in literature.

*Burkholderia cepacia* complex *(Bcc)* bacteria are opportunistic pathogens that can cause severe lung infections in immunocompromised people, including patients with cystic fibrosis (CF) [18]. Infections with *Bcc* bacteria are often difficult to treat due to resistance to many antibiotics. Moreover results from our group indicate that most *Bcc* biofilms contain a significant fraction of persister cells that can survive treatment with high doses of antibiotics [19]. In cells surviving treatment with high concentrations of tobramycin (4 x MIC), several genes encoding proteins involved in the generation of ROS, including enzymes of the tricarboxylic acid cycle or the electron transport chain were downregulated suggesting that in these surviving cells the production of ROS is lowered.

In the present study direct and indirect methods to measure the production of ROS in *Bcc* bacteria upon exposure to antibiotics were compared. Both planktonic and biofilm cultures were studied and antibiotics belonging to different classes (tobramycin, ciprofloxacin, meropenem) were included.

## Material and Methods

### Strains and culture conditions

The strains used in this study are listed in Table 1. Strains were cultured at 37°C on Luria-Bertani agar (LBA, Oxoid, Hampshire, UK). Overnight cultures were diluted in Luria-Bertani broth (LBB, Oxoid) and incubated aerobically at 37°C. *B*. *cenocepacia* K56-2 containing an *oxyR*::*lux* promoter fusion was grown on LBA supplemented with 100 μg/ml trimethoprim (Tp) (Ludeco, Brussels, Belgium) or in LBB supplemented with 100 μg/ml Tp [20, 21].

**Table 1:** Strains used in this study.

Strain	Strain info	Source (reference)
*B*. *cenocepacia*		
J2315 (LMG16656)	CF patient, UK	BCCM/LMG Bacteria Collection
K56-2 (LMG18863)	CF patient, Canada	BCCM/LMG Bacteria Collection
*oxyR*	K56-2P_oxyR_::pGSVTp-luxCDABE,Tp^r^	Prof. Valvano [21]
C5424 (LMG18827)	CF patient, Canada	Prof. Valvano [22]
MDL2	C5424 Δ*katB*	Prof. Valvano [22]
Triple quorum sensing deletion mutant	J2315 Δ*cepI*Δ*cciI*ΔBCAM0581	Prof. Riccardi [23]
*B*. *cepacia* LMG1222	*Allium cepa*, US	BCCM/LMG Bacteria Collection
*B*. *multivorans* LMG13010	CF patient, Belgium	BCCM/LMG Bacteria Collection
*B*. *vietnamiensis* LMG10929	Rice, soil, Vietnam	BCCM/LMG Bacteria Collection
*B*. *metallica* LMG24068	CF patient, US	BCCM/LMG Bacteria Collection

Tp^r^, trimethoprim resistance.

### Determination of the Minimal Inhibitory Concentration (MIC)

MICs were determined in duplicate according to the EUCAST broth microdilution protocol using flat-bottom 96-well microtiter plates (TPP, Trasadingen, Switzerland) [24]. Tobramycin (Sigma-Aldrich) concentrations tested ranged from 0.25 to 1024 μg/ml, ciprofloxacin (Sigma-Aldrich) and meropenem (Astrazeneca, The Netherlands) concentrations from 0.25 to 128 μg/ml. Mannitol (Sigma-Aldrich) concentrations tested ranged from 0.4 to 200 mM, glutathione (Sigma-Aldrich), cysteine concentrations (Sigma-Aldrich) from 0.002 to 1%, and DMSO (Sigma-Aldrich) concentrations from 0.008 to 4%. The pH of the antioxidant solutions was adjusted to 7.4. Solutions were freshly prepared and filter sterilized. The MIC was defined as the lowest concentration for which no significant difference in optical density (λ = 590 nm) was observed between the inoculated and blank wells after 24 h of incubation. All MIC determinations were performed in duplicate. Results obtained in replicate experiments never differed more than two fold; when a twofold difference was observed between replicates, the lowest concentration was recorded as the MIC.

### Planktonic growth

For the planktonic experiments an overnight culture was diluted to an optical density of 0.1 (approximately 10^8^ cells/ml). After an additional 24 h of growth in a shaking warm water bath cell suspensions with an optical density of 1 (approximately 10^9^ cells/ml) were transferred to falcon tubes and centrifuged for 9 min at 3634 rcf. Cells were resuspended in fresh medium or antibiotic solutions and further incubated at 37°C.

### Biofilm formation

Biofilms were grown as described previously [19]. Briefly, 100 μl of an overnight culture containing approximately 5 x 10^7^ CFU/ml was added to the wells of a round-bottomed 96-well microtiter plate (TPP) or for fluorescence or luminescence measurement, a flat-bottomed black 96-well microtiter plate (Perkin Elmer). Following 4 h of adhesion, the supernatant was removed and the plates were rinsed with physiological saline (0.9% w/v NaCl) (PS). Subsequently, 100 μl of fresh LBB was added and the plates were further incubated at 37°C. After 24 h, the supernatant was removed and fresh medium or an antibiotic solution was added. At least 6 wells were included per condition. Plates were further incubated at 37°C.

### Fluorometric determination of ROS

To measure differences in ROS production between treated and untreated cultures, 24 h old biofilms or planktonic cultures were exposed to 2',7'-dichlorodihydrofluorescein diacetate (H2DCFDA) or hydroxylphenyl fluorescein (HPF) in a final concentration of 10 μM and 5 μM, respectively, in LBB. After 45 min of incubation protected from light cells were washed with PBS and treated with H_2_O_2_, tobramycin (Tob), ciprofloxacin (Cip), meropenem (Mer) or pH-matched phosphate buffered saline (PBS) (= untreated control solution with the same pH as the antibiotic solution). When cultures were treated with H_2_O_2_ concentrations up to 3% were used and cells were treated for 30 min. The antibiotic concentrations ranged from 0.25 x MIC to 16 x MIC and cells were treated up to 24 h. Fluorescence (λ excitation = 485 nm, λ emission = 535 nm) was measured using an Envision plate reader. Autofluorescence of bacterial cells incubated without the probe and background fluorescence of the buffer solutions was measured and taken into account when calculating the net fluorescence. 3–6 wells were included per condition and each experiment was repeated twice (n = 3 x 3 or 6).

### Measuring the expression of OxyR

To measure the expression of OxyR, a marker of oxidative stress, *B*. *cenocepacia* K56-2 carrying an *oxyR*::*lux* promoter fusion was used [21]. Biofilms were grown in black microtiter plates in LBB supplemented with 100 μg/ml Tp. After 24 h of growth the supernatant was removed and cells were treated with Tob (2xMIC), Cip (4xMIC), Mer (4xMIC), H_2_O_2_ (0.03%) or LBB (control). For the planktonic experiments 24 h old cultures with an optical density of 0.5 were transferred to falcon tubes and centrifuged for 9 min 3634 rcf. Cells were resuspended in an antibiotic solution in LBB and transferred to the wells of a black 96-well microtiter plate (200 μl per well). Luminescence was measured over time using an Envision microtiter plate reader. Six wells were included per condition and each experiment was repeated twice (n = 3 x 6).

### EPR

Planktonic cultures of *B*. *cenocepacia* K56-2 were grown as described above. After 24 h of growth cultures were treated with Tob (4 x MIC), Cip (4 x MIC), Mer (4 x MIC) or PBS (control). 30 min before the designated time points, samples were taken and mixed (1:1) with a superoxide-specific spin probe solution containing 10 mM 1-Hydroxy-3-methoxycarbonyl-2,2,5,5-tetramethylpyrrolidine (CMH) (Noxygen Science Transfer & Diagnostics GMBH, Germany) dissolved in Krebs-Hensleit buffer (Noxygen Science Transfer & Diagnostics GMBH, Germany). Each sample was incubated for an additional 30 minutes at 37°C, allowing superoxide to react with the probe. 60 μL of this solution was subsequently loaded into a quartz capillary, which was placed in the resonator cavity of the EPR spectrometer. The spectra were collected at a constant room temperature of 25°C at a center field of 3350 G with a sweep of 100 G on a MS 200 spectrometer (Magnettech, Germany). Resulting spectra were recorded and analyzed as described previously [25] using the software package Analysis 2.02 (Magnettech, Germany) (n = 4 x 2). Solutions of Tob, Cip and Mer in PBS and 100% PBS in the absence of bacteria were included as controls.

### Effect of the addition of antioxidants on metabolism of *B*. *cenocepacia* K56-2 biofilms

To determine whether the antioxidants had an influence on metabolism, biofilms were grown as described above. After 24 h, the supernatant was removed, and 100 μl of an antioxidant solution in PS was added to the wells. After 30 min incubation at 37°C the supernatant was removed and 120 μl of a commercially available resazurin solution (CellTiter-Blue, Promega, Madison, WI, USA) was added to all wells. Fluorescence (λ excitation = 560 nm, λ emission = 590 nm) was measured after 2 h of incubation. Twelve wells were included per condition.

### Effect of the addition of antioxidants on survival of *B*. *cenocepacia* K56-2 biofilms and planktonic cultures

To determine the effect of adding antioxidants to an antibiotic treatment, antioxidant solutions in PS were added before treatment. After 30 min of pre-incubation an antibiotic solution in PS was added and cultures were incubated for an additional 24 h. The antioxidants tested were glutathione (15 mM), mannitol (100 mM), cysteine (0.25%), PDTC (0.1 mM) and sodium pyruvate (20 mM). The pH of the antioxidant solutions was adjusted to 7.4. The final antibiotic concentration was 4 x MIC. After 24 h of treatment, biofilm cells were washed with PS and harvested by vortexing and sonication (2 x 5 min) (Branson 3510, Branson Ultrasonics Corp, Danbury, CT). Planktonic cultures were centrifuged (5 min, 3634 rcf) and resuspended in PS. Cells were quantified by plating on LBA (n ≥ 3 for all experiments).

### Determination of survival after treatment

To determine the number of surviving cells, 24 h old biofilms and stationary phase planktonic cultures were exposed to Tob, Cip or Mer in a concentration of 4 x MIC for 24 h [19]. Biofilms and planktonic cultures were grown as described above. After 30 min, 2 h, 6 h and 24 h of growth in a biofilm, the supernatant was removed and 120 μl of an antibiotic solution in PS or 120 μl PS (= control) was added and the plates were incubated for an additional 24 h at 37°C. Twelve wells were included per condition. Cells were harvested by vortexing and sonication (2 x 5 min) (Branson 3510, Branson Ultrasonics Corp, Danbury, CT) and quantified by plating on LBA (n ≥ 3 for all experiments). For the planktonic experiments, stationary phase cultures with an optical density of 1 were centrifuged and resuspended in PS or an antibiotic solution in PS. After 30 min, 2 h, 6 h and 24 h of treatment the number of surviving cells was quantified by plating on LBA (n ≥3 for all experiments).

### Detection of protein carbonylation and formation of 8-hydroxydeoxyguanosine

To study whether exposure to antibiotics had an effect on protein carbonylation and formation of 8-hydroxydeoxyguanosine, planktonic cultures of *B*. *cenocepacia* K56-2 (OD = 1) were treated with Tob (4 x MIC), Cip (4 x MIC), Mer (4 x MIC), H_2_O_2_ (3%) or LB (= control). Cells were treated with H_2_O_2_ or antibiotic solutions for 30 min or 24 h, respectively. To measure protein carbonylation 1 ml of the suspension was transferred to a sterile microcentrigue tube. The microcentrifuge tubes were centrifuged for 10 minutes at 20817 rcf. The supernatant was removed, pellets were washed with 50 mM Tris and resuspended in 150 μl lysis buffer (5 mg/10 ml lysozyme, 1 mM EDTA, 10 mM Tris) and 15 μl of a 10% sodium dodecyl sulfate solution. After 5 minutes at 100°C the microcentrigue tubes were centrifuged for 5 min at 10 621 rcf and 4°C and the supernatant was transferred to a new microcentrifuge tube. Protein concentration and carbonylation was determined immediately following the extraction using the Bicinchoninic Acid (BCA) kit (Sigma-Aldrich) and the Oxiselect protein carbonyl fluorometric kit (Bio-Connect, The Netherlands) respectively, according to the manufacturer’s instructions. To quantify 8-hydroxydeoxyguanosine formation 50 ml of each suspension was centrifuged for 6 min at 3634 rfc. The resulting pellet was resuspendend in 1 ml PS and transferred to a sterile microcentrifuge tube. The tubes were centrifuged (10 min, 17 949 rcf) and DNA was extracted as described previously [26]. Two samples were pooled per condition and the 8-OHdG concentration was determined using the Oxiselect Oxidative DNA damage ELISA kit (Bio-Connect) according to the manufacturer’s instructions.

### Statistical data analysis

Statistical data analysis was performed using SPSS software, version 21 (SPSS, Chicago, IL, USA). The Shapiro-Wilk test was used to verify the normal distribution of the data. Normally distributed data were analysed using a one-sample T-test or an independent sample T-test, whereas non-normally distributed data were analysed using a Wilcoxon signed-rank test or a Mann-Whitney test. P-values < 0.05 were considered significant.

## Results and Discussion

### Comparison of different methods to measure the production of ROS upon antibiotic treatment

To compare different ROS detection methods, both planktonic and biofilm cultures were studied and antibiotics belonging to different classes (Tob, Cip, Mer) with different killing effects on *Bcc* species were included. Tob has the highest bactericidal activity against *Bcc* bacteria. After 2 h 96% and 93% of the cells are killed in biofilm or planktonic culture, respectively; after 24 h, we observed a 4–5 log reduction in the number of suriviving cells (S1 Fig). Mer has no significant bactericidal effect on *Bcc* species when grown planktonically (S1 Fig). In biofilm cultures still 23% of the cells are alive after 24 h of treatment. For Cip a 50% reduction is observed after 2 h and after 24 h 98 and 89% of the cells are killed in biofilms and planktonic cultures, respectively (S1 Fig).

#### Detection of ROS using fluorescein based stainings

To determine whether antibiotics induce the generation of ROS in *Bcc* species we used two fluorescent reporter dyes, 2’,7’-dichlorodihydrofluorescein diacetate (H2DCFDA) and hydroxyphenylfluorescein (HPF), to directly measure ROS production. These dyes have already been used in previous studies, but their use has recently been the subject of considerable debate [5, 9]. In the present study we investigated whether they can be used to measure ROS production in *Bcc* bacteria.

**Fig 1:**
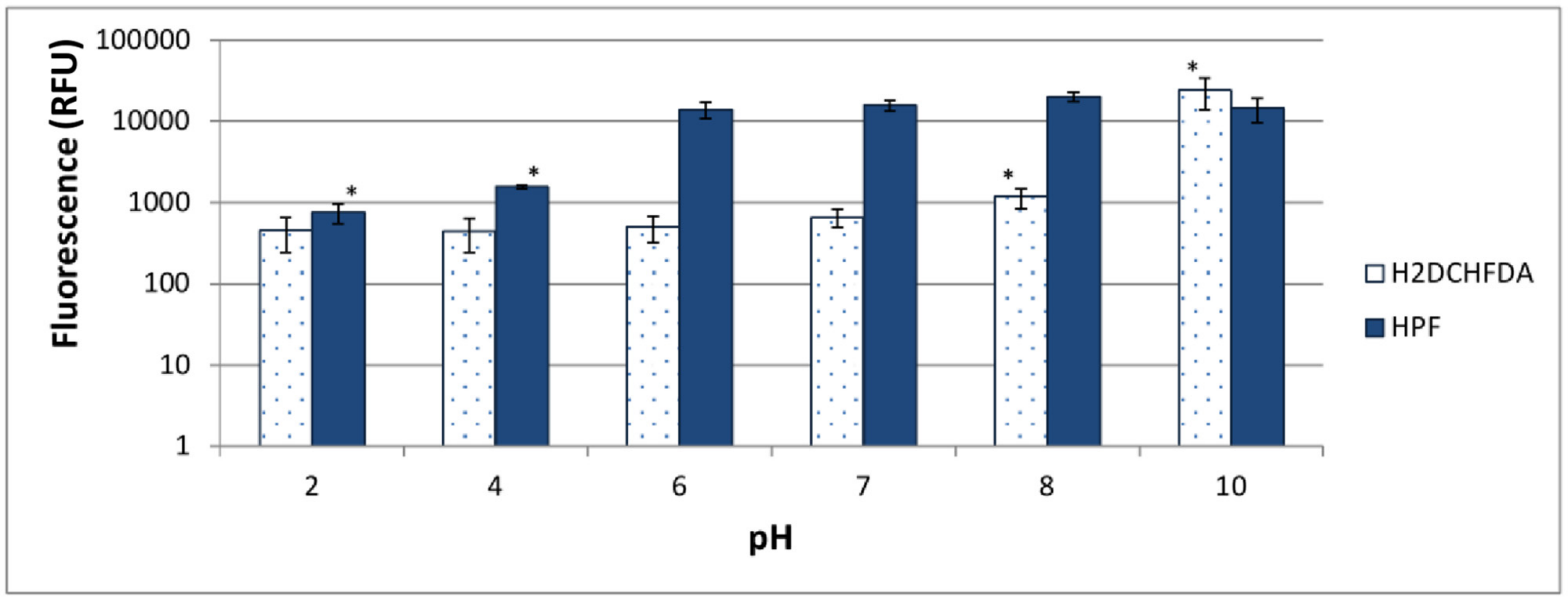
Influence of pH on fluorescence of H2DCFDA and HPF. Error bars represent SEM. Solutions for which fluorescence was significantly different from fluorescence at pH 7 are indicated with an asterisk, p < 0.05, n = 3.

Since *Bcc* species are highly resistant against antibiotics [27], high concentrations are needed. However, fluorescein based approaches for ROS quantification are pH sensitive and such high concentrations of antibiotics have an influence on the pH of the solution. For example, a Tob solution of 1024 μg/ml in PBS has a pH of approximately 9. Therefore we first tested the influence of pH on the fluorescence of H2DCFDA and HPF. Fluorescence of H2DCFDA and HPF was found to be highly pH dependent (Fig 1), so we decided to use a protocol in which cultures are first pre-incubated with the dye and are only exposed to the antibiotic after removal of the dye. This way the results cannot be biased by differences in pH. Another advantage of this approach is that differences in fluorescence cannot be attributed to differences in uptake of the dye between treated and untreated cells. Antibiotics may disrupt the cytoplasmic membrane and as suggested by Imlay et al (2015) disruption of the membrane barrier can affect the amount of the dye that penetrates into cells and can thus influence fluorescence [7].

**Fig 2:**
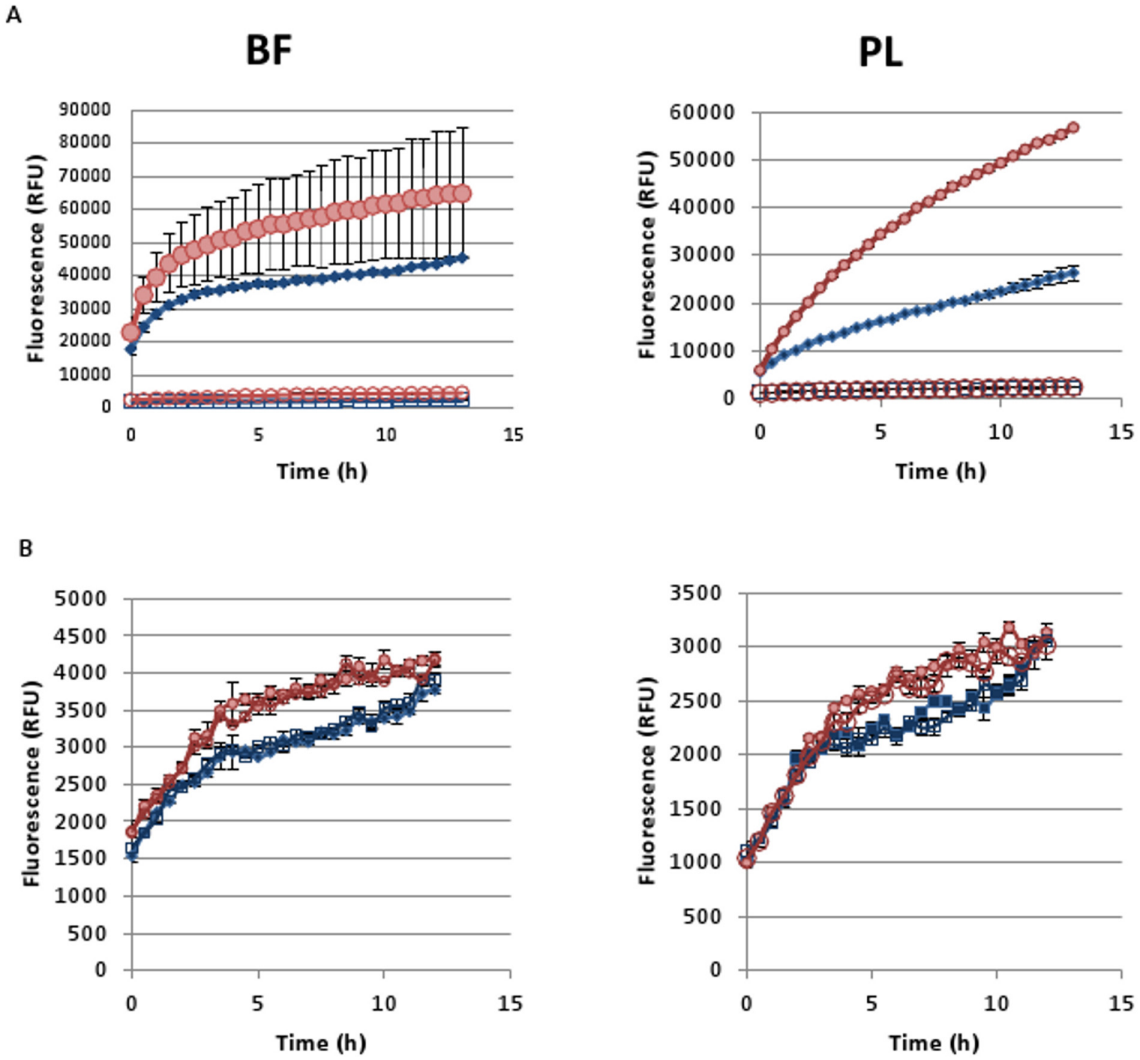
Fluorescence generated over time in treated (4 x MIC, up to 13 h) (red circles) and untreated (blue squares) biofilms (left) and planktonic cultures (right). (A) Cultures incubated with (closed circles and squares) or without (open circles and squares) H2DCFDA. (B) Cultures incubated with (closed circles and squares) or without (open circles and squares) HPF. Data are shown of a single representative experiment. Error bars represent SEM (calculated on 3 technical replicates).

Next to differences in pH, also differences in autofluorescence have been described in literature as a confounding factor [9, 28]. Therefore autofluorescence was measured and subtracted from the total fluorescence values. However, in contrast to what is described in the literature for exponentially growing *E*. *coli* cultures [9, 28], there were no significant differences in autofluorescence between the treated and untreated stationary phase planktonic *B*. *cenocepacia* cultures. In biofilms autofluorescence was slightly higher after treatment, but for H2DCFDA the autofluorescence values were very low compared to the total fluorescence values, indicating our results are not biased by differences in autofluorescence (Fig 2A). HPF, on the other hand was not used in further experiments since autofluorescence values were high compared to the total fluorescence values (Fig 2B).

Using our protocol almost a 2-fold increase in H2DCFDA fluorescence was observed when biofilms or planktonic cultures were treated with Tob in a concentration of 4 x MIC compared to untreated cultures (Fig 2A), with more variation between biofilm replicates. As a positive control biofilms and planktonic cultures were treated with different concentrations of H_2_O_2_ (up to 3% = 882.4 mM). For H_2_O_2_ a 3- and 5-fold difference between treated and untreated biofilms and planktonic cultures was observed, but there was no linear relationship between the increase in fluorescence and the H_2_O_2_ concentration tested (S2 Fig).

These results suggest that our method can be used to measure ROS production upon antibiotic treatment. To confirm that H2DCFDA can be oxidized by intracellularly produced ROS, fluorescence was measured in a *B*. *cenocepacia* catalase deletion mutant (Δ*katB*). Fluorescence was indeed higher (p < 0.05) in the mutant compared to in the wild type before (2.8 fold) and after treatment with Tob (2.1 fold) confirming that the dye can be oxidized by intracellularly produced ROS (S3A Fig). The expression of *katB* is positively regulated by CepR and similarly fluorescence was more than two-fold (p < 0.05) higher in a triple quorum sensing mutant (S3B Fig).

Together, these results suggest that using the appropriate controls, antibiotic-induced ROS production in the *Bcc* can be measured using fluorescein based stainings. As variability between replicates can be quite high, care should be taken when results obtained in different experiments are compared.

#### The induction of OxyR

**Fig 3:**
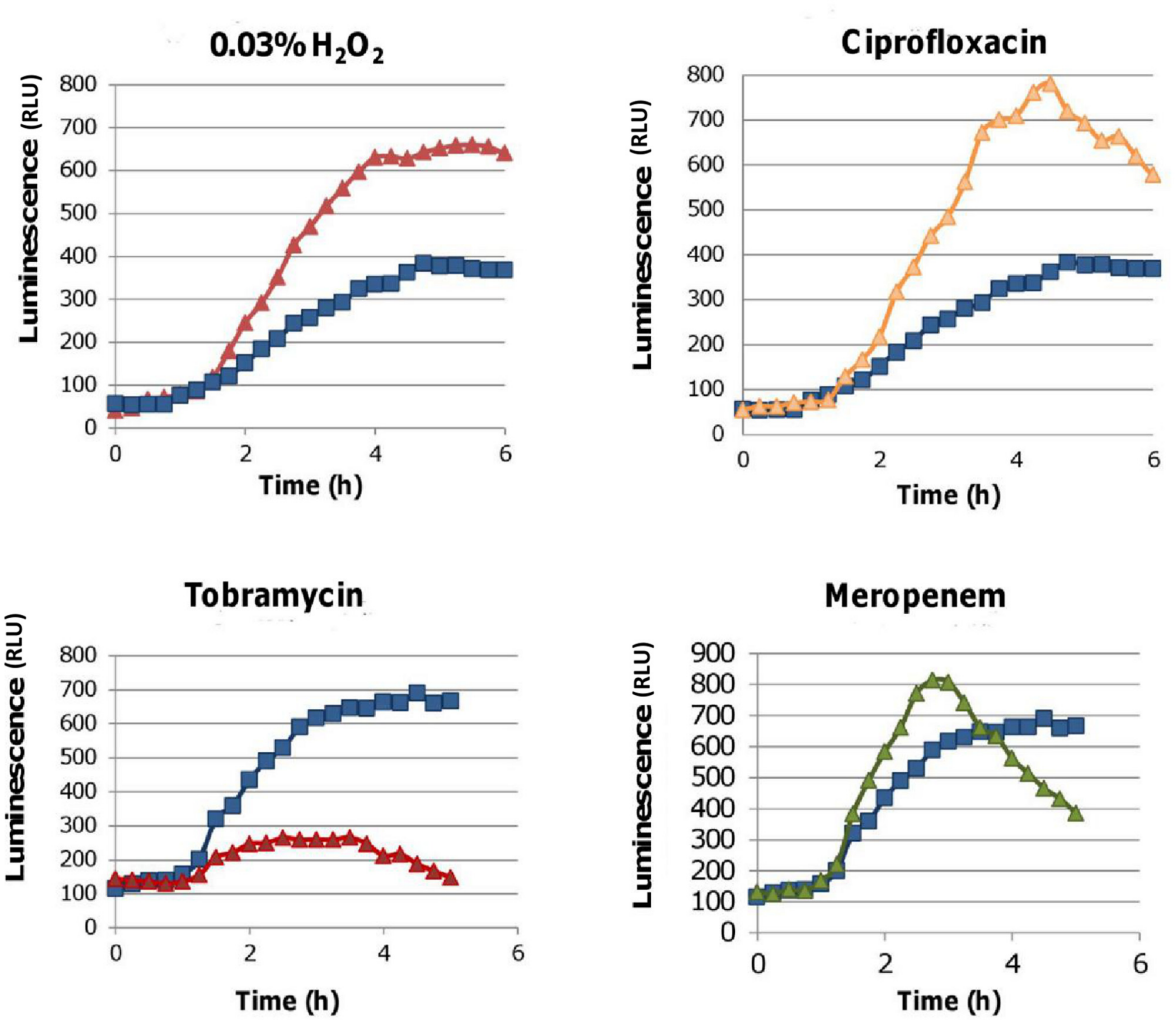
Luminescence generated over time after treating a *B*. *cenocepacia* K56-2 *oxyR*:*lux* promoter fusion mutant biofilm with 0.03% H_2_O_2_, Cip (4 x MIC), Mer (4 x MIC) or Tob (2 x MIC) (up to 6 h) compared to luminescence in biofilms exposed to LB alone (blue). Data are shown of a single representative experiment.

A more specific way to measure ROS is looking at the induction of *oxyR* [29]. OxyR is a transcription factor by which a cell senses threatening levels of H_2_O_2_. To measure the induction of OxyR, an *oxyR*::*lux* promoter fusion was used [21]. We found that the addition of 0.03% H_2_O_2_ or Cip considerably increased luminescence both in biofilms (Fig 3) and planktonic cultures (S4 Fig). The observed differences were time and concentration dependent, with no effect for very low or high concentrations. A small difference was also observed after treatment with Mer. While this is a sensitive method some considerations should be taken in to account. For example, this method cannot be used with aminoglycosides which inhibit protein synthesis and antibiotic solutions should be prepared in growth medium, since a functional translational machinery is necessary to produce the lux proteins.

#### Electron Paramagnetic Resonance

A specific way to directly measure the production of ROS without the need of functional translation is Electron Paramagnetic Resonance (EPR) [30]. Since free radicals have short half-lives, EPR is used in combination with spin traps or spin probes to yield relatively long-lived and detectable radical adducts. In the present study CMH, a spin probe that quickly reacts with superoxide radicals was used.

**Fig 4:**
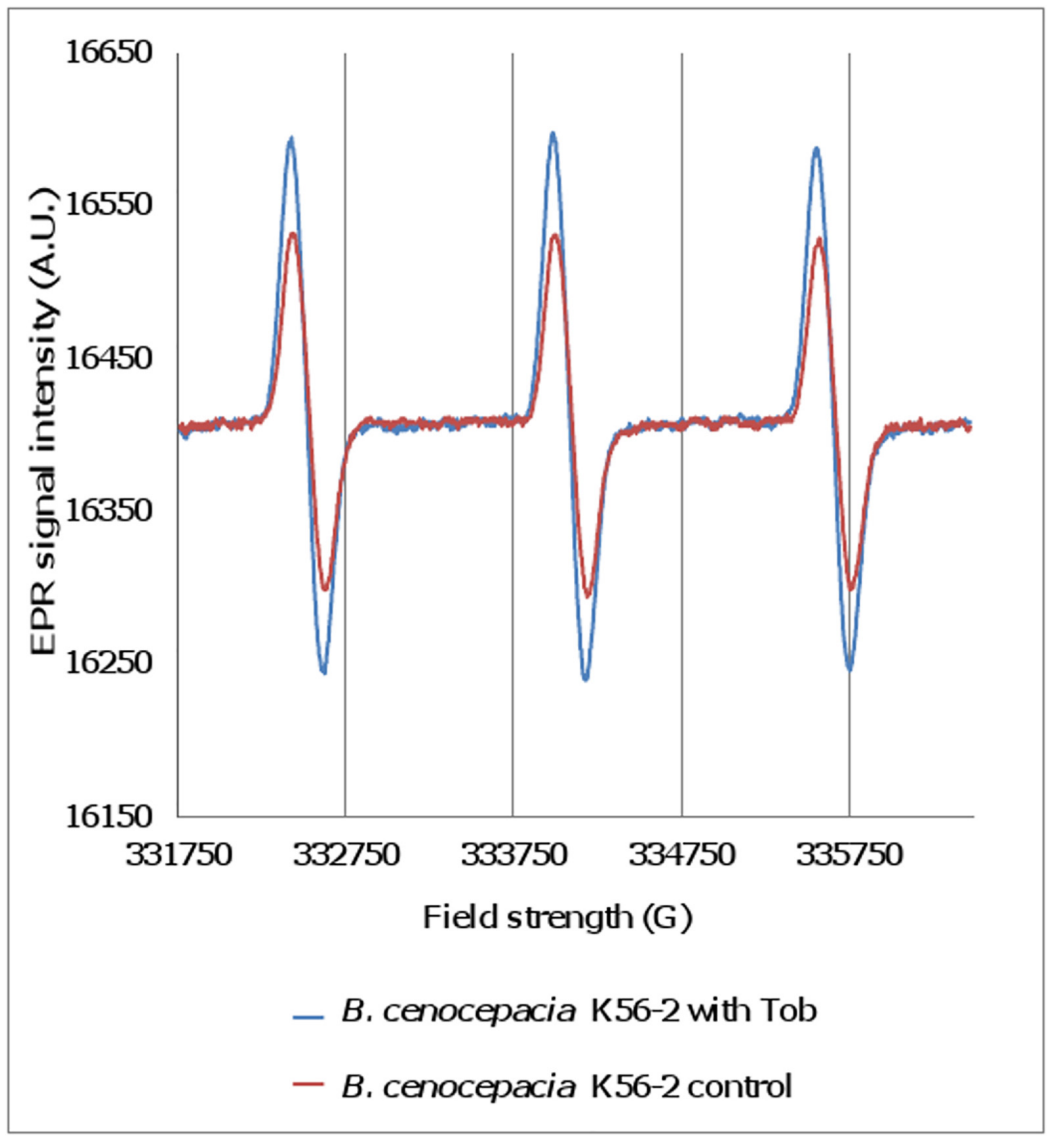
EPR spectrum of the spin probe CMH and superoxide radicals formed in planktonic cultures of *B*. *cenocepacia* K56-2 treated with Tob (4 x MIC) for 30 min. Data are shown of a single representative experiment.

**Fig 5:**
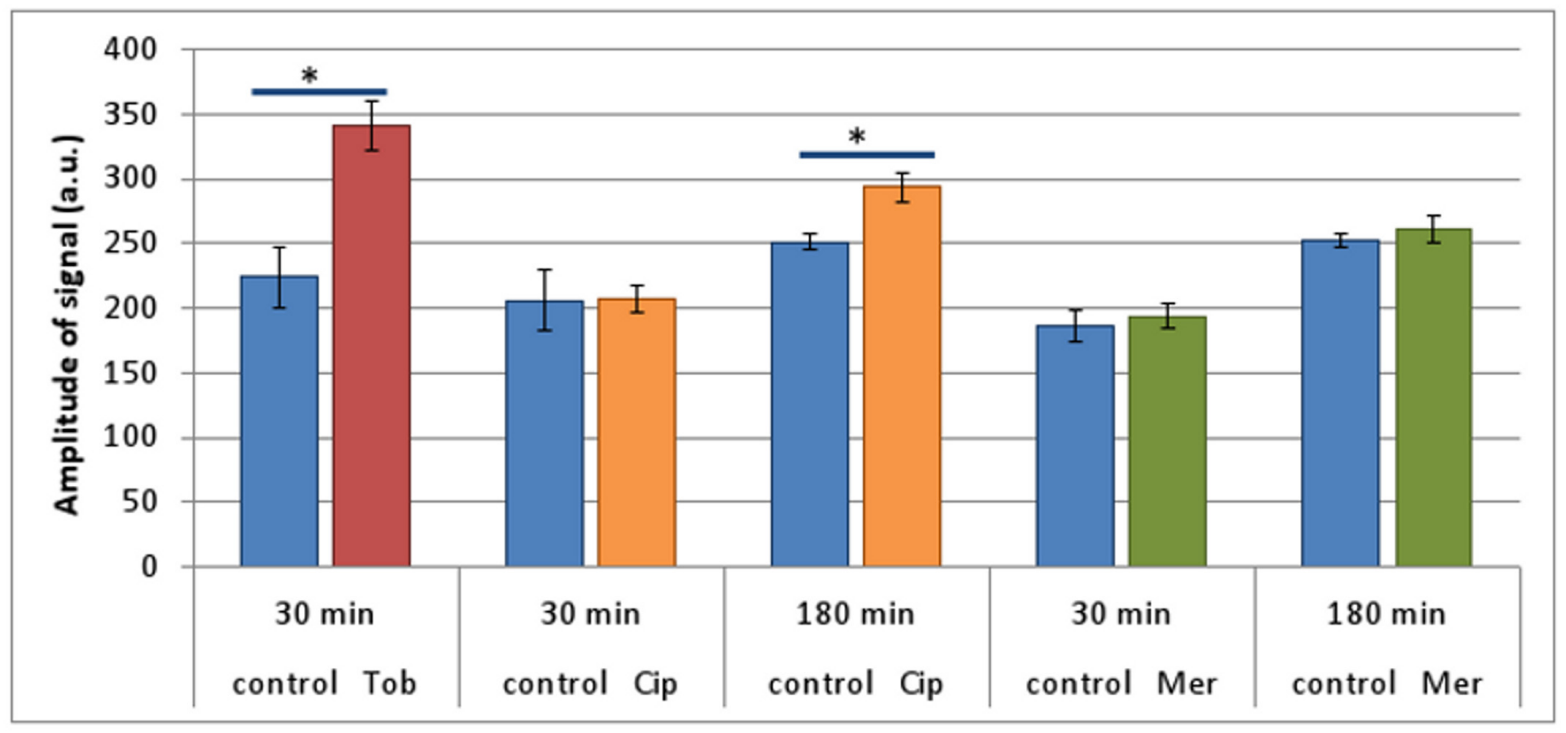
EPR determination of radicals formed in treated and untreated (blue) planktonic cultures of *B*. *cenocepacia* K56-2. Cultures were treated with Tob (4 x MIC, 30 min, red), Cip (4 x MIC, 30 min and 180 min, orange) or Mer (4 x MIC, 30 min and 180 min, green). The probe was added 30 min before measurement. Error bars represent SEM. Statistically significant differences are indicated with an asterisk, p < 0.05, n = 4.

When Tob-treated (30 min) planktonic cultures of *B*. *cenocepacia* K56-2 were investigated, a characteristic spectrum confirming the presence of superoxide radicals was observed (Fig 4). The amplitude of the signal was higher in cultures treated with Tob than in untreated cultures (Fig 5) but differences were only significant after 30 min of treatment. It is likely that the amplitude of the signal decreases over time in the Tob treated cultures, because of cells being killed by the antibiotic (S1 Fig). This observation illustrates the importance of measuring ROS production at different time points. For Cip the amplitude of the signal was only higher in treated cultures (compared to untreated ones) after 180 minutes of treatment (Fig 5) which is in agreement with the increase in luminescence observed 2 h after treatment with Cip using the *oxyR*::*Lux* promoter fusion strain. No significant increase in ROS production was measured after treatment with Mer for 30 or 180 min.

#### Survival after treatment with antibiotics in combination with antioxidants

**Fig 6:**
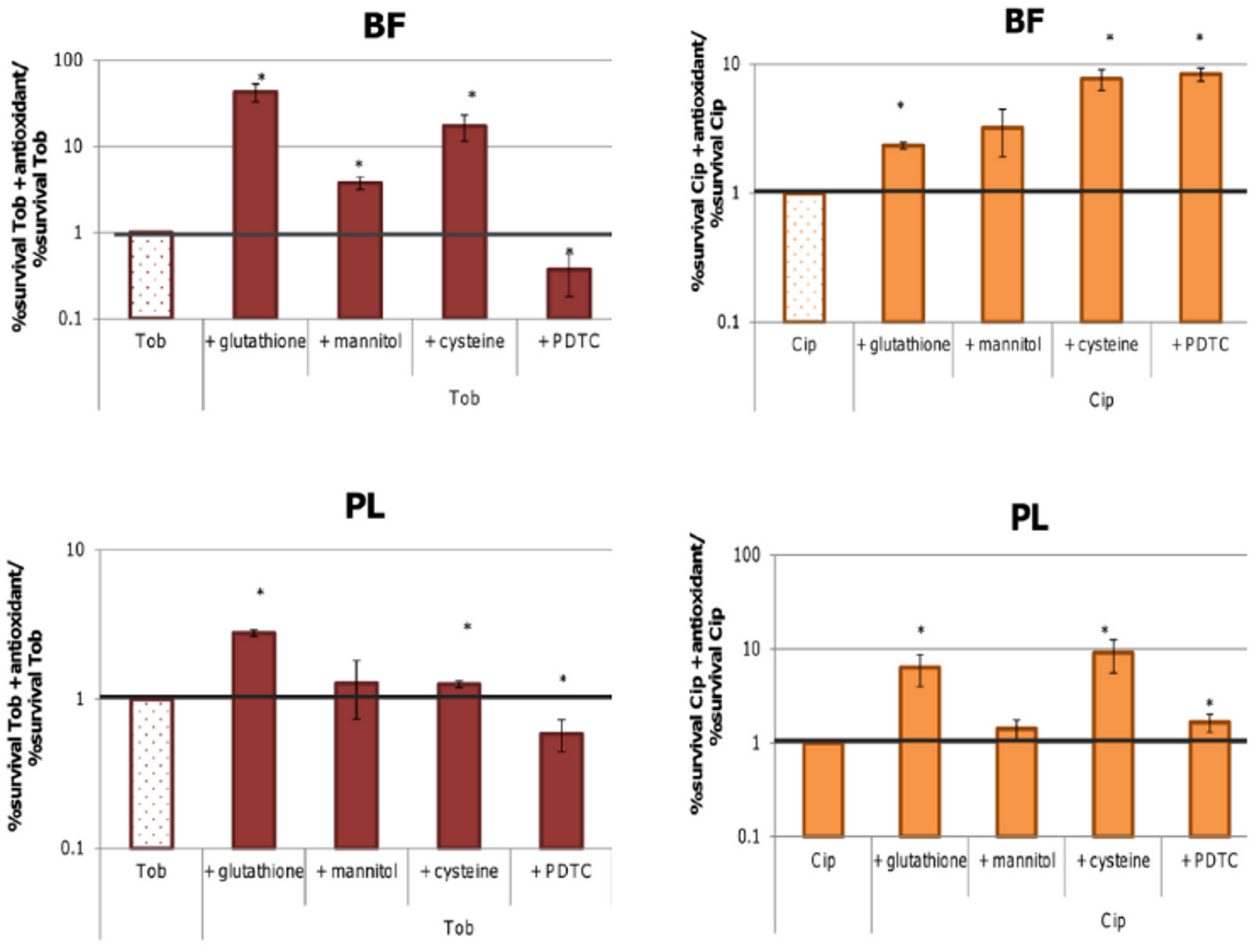
The fraction of surviving cells after treating biofilms (top) or planktonic cultures (bottom) with Tob or Cip (4 x MIC, 24 h) in combination with an antioxidant, compared to the fraction surviving cells after treatment with Tob or Cip alone. Error bars represent SEM. Statistically significant differences are indicated with an asterisk, p < 0.05, n = 3.

An indirect method to measure ROS production is measuring the influence of adding antioxidants. A broad range of antioxidants was tested in a concentration well below their MIC (S1 Table). The pH of the solutions was adjusted to 7.4 and the influence on metabolism was tested. For the antioxidants included in this study no negative influence on metabolism was observed (S5 Fig), but generally these antioxidants had a positive influence on survival (Fig 6).

**Fig 7:**
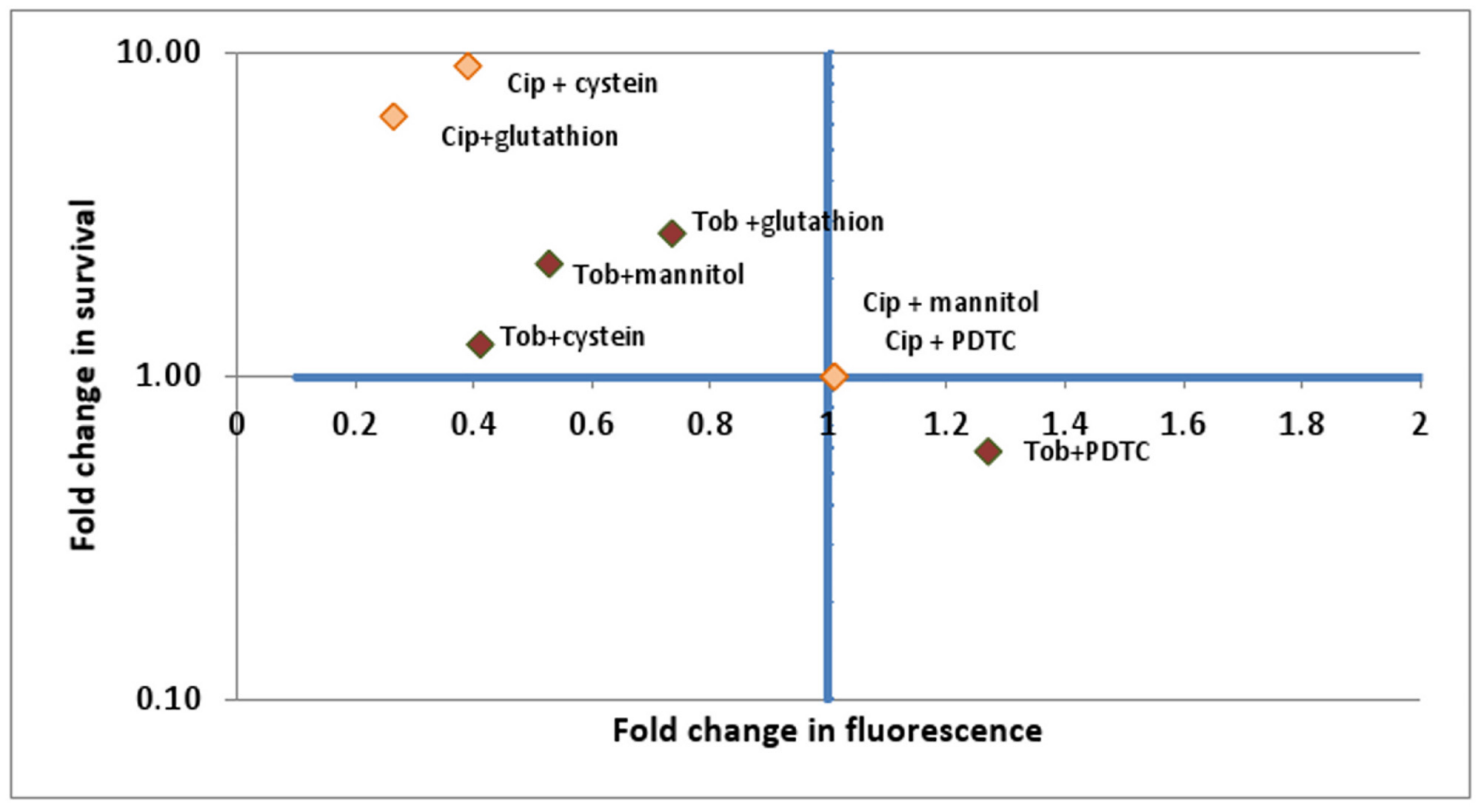
Correlation between fold change in survival and fold change in fluorescence after treatment with Tob or Cip (4 x MIC, 24 h) in combination with an antioxidant compared to treatment with Tob or Cip alone.

To investigate whether the protective effect of the antioxidants effectively correlates with a decrease in ROS production, H2DCFDA fluorescence was measured over time after treating planktonic cultures with Tob or Cip in combination with one of the antioxidants. Generally there is a good correlation between the influence on fluorescence and survival, with lower ROS levels correlating with increased survival and vice-versa (Fig 7).

Subsequently, we also investigated whether there is a correlation between the protective effects of the antioxidants and the expression of OxyR. Biofilms were pre-incubated with one of the antioxidants and treated with Cip. Luminescence was measured over time for 6 h. Unexpectedly, addition of an antioxidant generally did not decrease the expression of OxyR. The expression of OxyR was only lower compared to with Cip alone when PDTC was added (S6 Fig). Surprisingly, similar results are also observed in combination with H_2_O_2_ (S7 Fig) suggesting that quantifying the expression of OxyR cannot be used to measure the effects of antioxidants on ROS production.

So while our initial results suggest that antioxidants increase survival by scavenging ROS, additional protective mechanisms are likely involved. For example, Goswami et al. (2007) found that glutathione protects against fluorochinolones and aminoglycosides but that only for chinolones the protective mechanism involves scavenging of ROS [13]. Similarly, Dhamdhere et al. (2010) found that in *E*. *coli* addition of glutathione also increased the MIC for erythromycin, a bacteriostatic antibiotic, which does not induce oxidative stress, suggesting protection by glutathione involves more than protection against oxidative damage alone [31].

#### Measuring oxidative damage

Since elevated intracellular levels of OH can damage DNA, lipids and proteins, another approach to study the involvement of ROS in antibiotic mediated killing is measuring ROS induced damage. For example, Wang et al. (2014) observed that the amount of protein carbonylation was almost 2-fold increased in stationary *E*. *coli* cultures treated with gentamycin [32]. In the present study we measured protein carbonylation in *B*. *cenocepacia* K56-2 planktonic cultures after treatment with Tob (4 x MIC, 24 h), Cip (4 x MIC, 24 h) or H_2_O_2_ (positive control, 3%, 30 min) using a fluorometric assay. However, no significant differences were observed between antibiotic treated and untreated samples (S8 Fig). In addition only a 1.5 fold (p > 0.05) increase in carbonylation was observed after treatment with H_2_O_2_ (S8 Fig), suggesting this method is not sensitive enough to detect antibiotic induced oxidative damage in *Bcc* bacteria that are highly resistant against oxidative stress [33].

A study by Foti et al. (2012) suggested that cell death following ROS-mediated killing is predominantly caused by specific oxidation of the guanine nucleotide pool [34]. Because of its low redox potential, guanine is particularly susceptible to oxidation and 8-oxo-deoxyguanine is potentially mutagenic because of its ability to form base pairs with cytosine and adenine [35]. However, no significant difference was observed in the concentration of 8-hydroxydeoxyguanosine after treatment with Tob (4 x MIC), Cip (4 x MIC) or H_2_O_2_ (3%).

**Fig 8:**
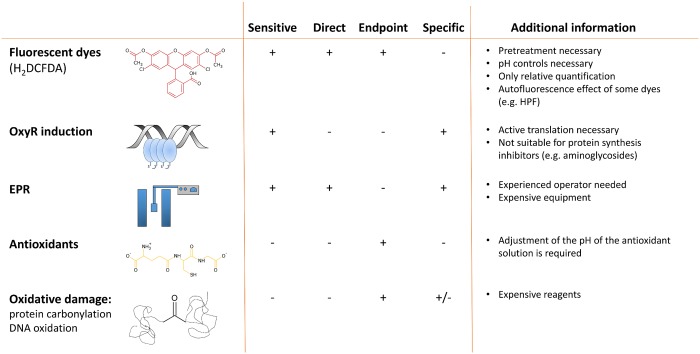
Summary of import aspects of ROS detection methods.

Overall these results highlight some methodological key issues to be considered when evaluating the contribution of ROS in antibiotic mediated killing. A summary of important aspects of the different methods is presented in Fig 8.

### Is ROS involved in antibiotic mediated killing in Bcc species?

**Fig 9:**
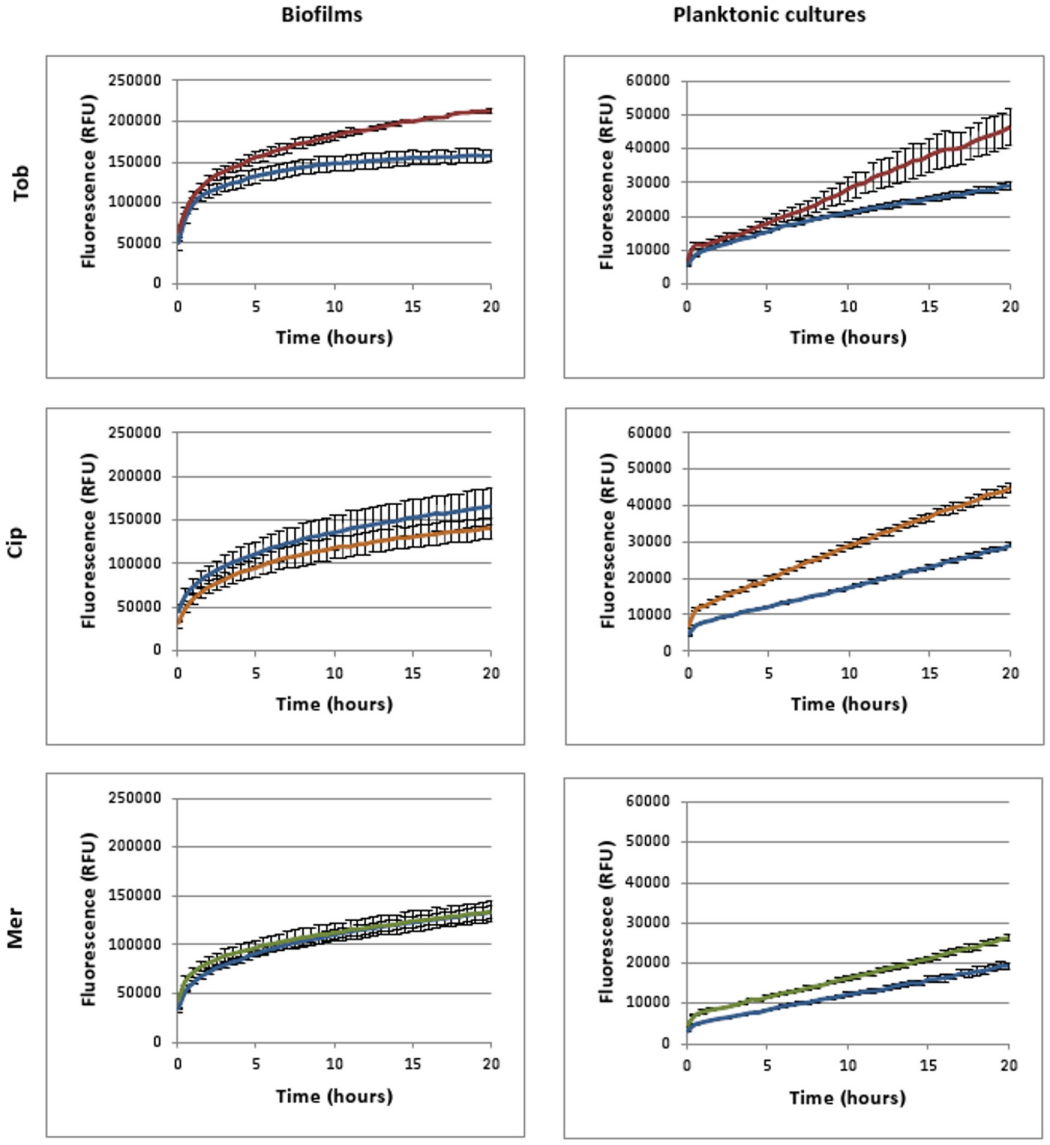
Fluorescence generated over time after treatment with Tob (4 x MIC, up to 20 h) (red), Cip (16 x MIC, up to 20 h) (orange) or Mer (16 x MIC, up to 20 h) (green) in biofilms and planktonic *B*. *cenocepacia* K56-2 cultures compared to fluorescence in pH-matched controls (blue). Cultures were pre-incubated with H2DCFDA and treated with antibiotics or control solutions with the same pH. Data are shown of a single representative experiment. Error bars represent SEM (calculated on 3 technical replicates).

**Fig 10:**
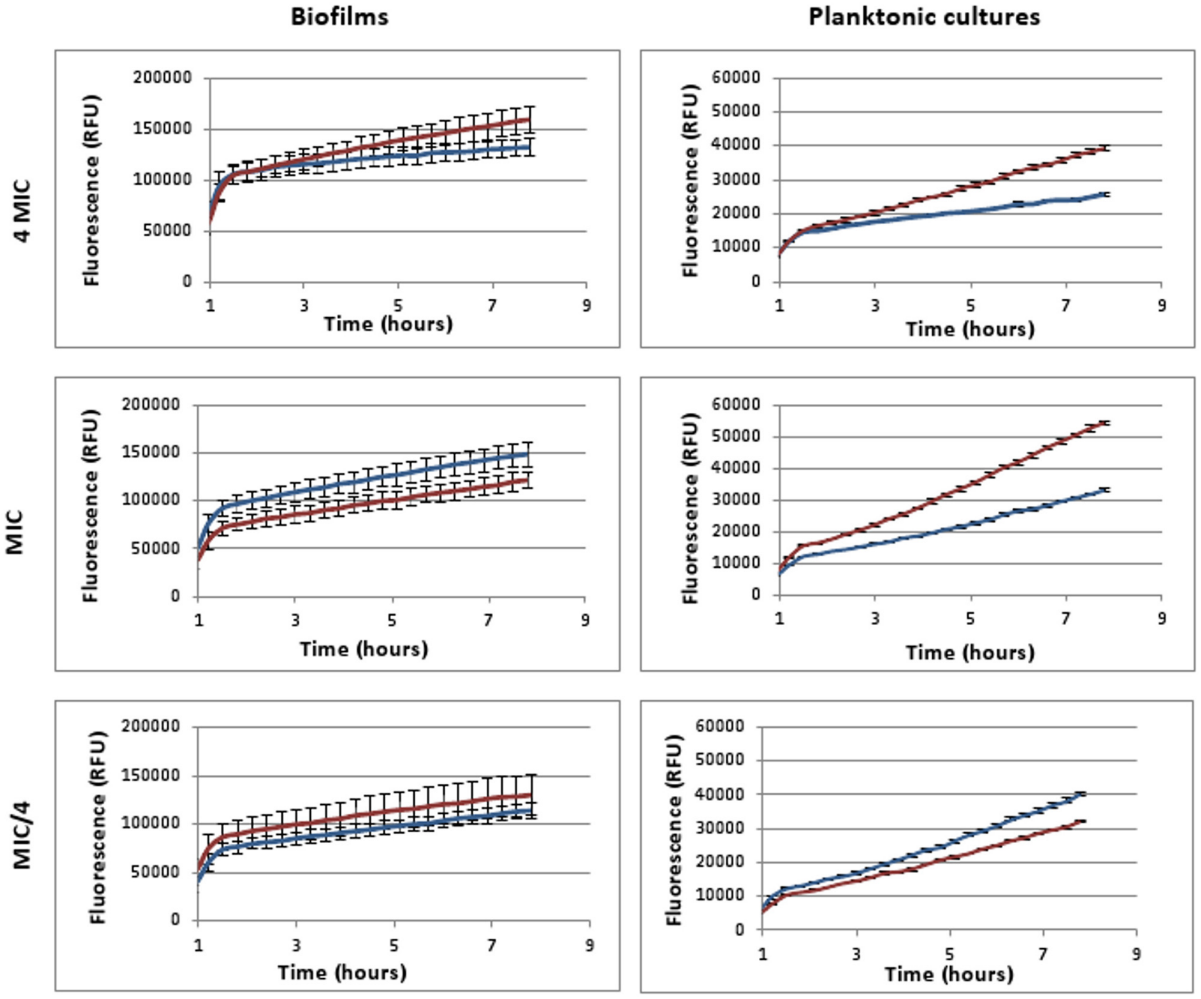
Fluorescence generated over time in biofilms and planktonic *B*. *cenocepacia* K56-2 cultures after treatment with Tob (red) in different concentrations (4xMIC, MIC, MIC/4, up to 8 h) compared to fluorescence in pH-matched control (blue). Cultures were pre-incubated with H2DCFDA and treated with antibiotics or control solutions with the same pH. Data are shown of a single representative experiment. Error bars represent SEM (calculated on 3 technical replicates).

In Fig 9 the fluorescence over time is shown in treated and untreated *B*. *cenocepacia* K56-2 biofilm and planktonic cultures after incubation with H2DCFDA. Cultures were treated with Tob (4 x MIC), Cip (16 x MIC), Mer (16 x MIC) or pH-matched control solutions. In the planktonic cultures fluorescence is higher after treatment with Tob or Cip whereas in biofilms fluorescence is only higher after treatment with Tob. For Mer differences were not statistically significant (p > 0.05). So an increased ROS production is measured for the antibiotics (Tob and Cip) that induce cell killing but the results seem highly dependent on the experimental conditions. ROS production is not only antibiotic dependent, it is also dependent on the time point at which fluorescence is measured. This is especially the case for biofilms, as only after 20 h a pronounced difference is observed. For Tob also different concentrations were tested (Fig 10). It is noteworthy that we only observed a higher relative increase in ROS production in treated vs. untreated conditions (~slope of the curves in Figs 9 and 10) in planktonic cultures treated with a Tob concentration of at least 1xMIC and in biofilms treated with at least 4xMIC. This difference after treatment with a concentration of 1xMIC between biofilms and planktonic cultures is likely due to the differences in cell killing. After 24 h, there is an additional 10-fold reduction in cells in planktonic cultures compared to biofilms [19]. Additionally there is inherently more variation between replicates in the biofilm assay, which may explain the lower fluorescence in the treated cultures compared to in the untreated cultures.

**Fig 11:**
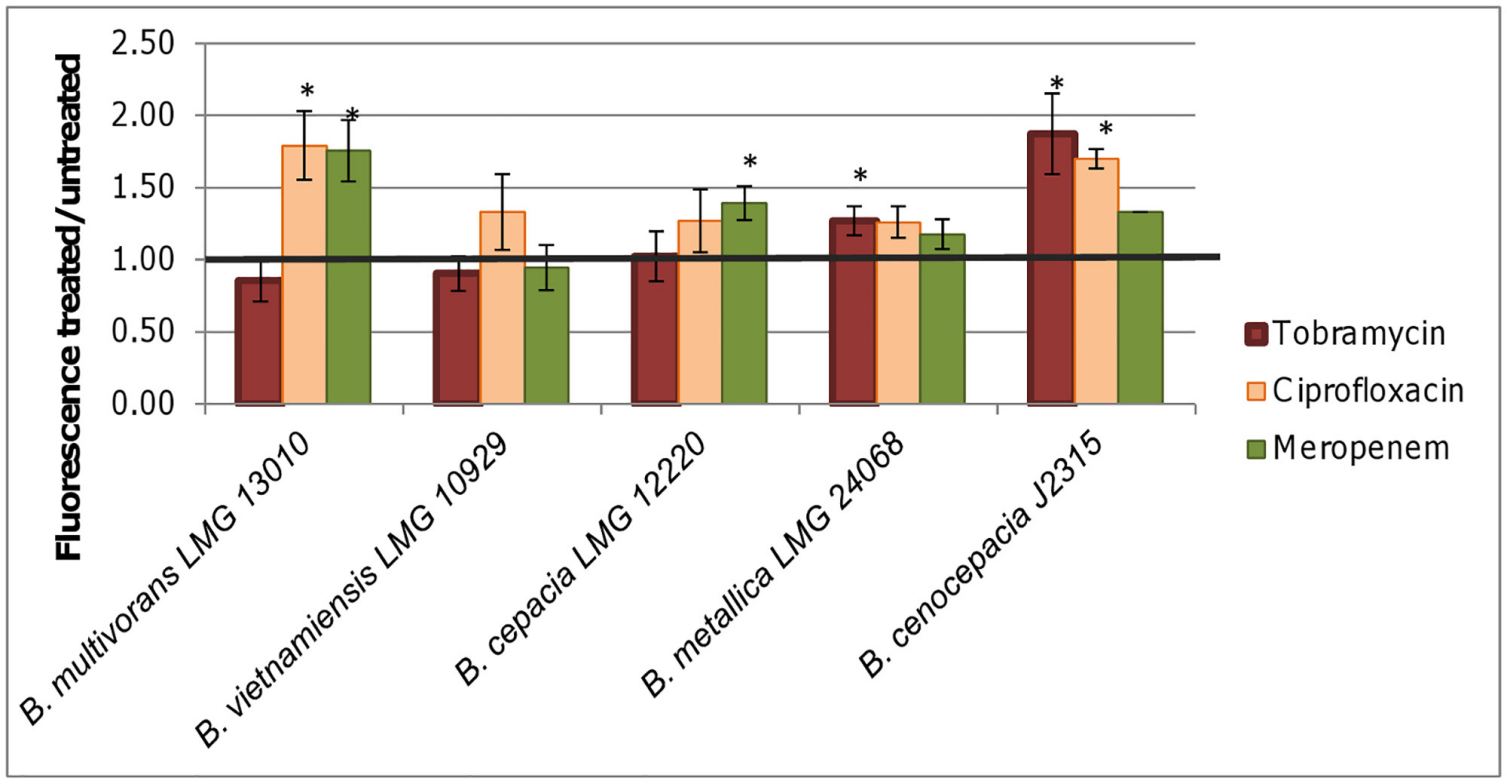
H2DCFDA fluorescence in treated (Tob 4 x MIC, Cip 16 x MIC, Mer 16 x MIC, 24 h) versus untreated planktonic cultures of *B*. *multivorans* LMG 13010, *B*. *vietnamiensis* LMG 10929, *B*. *cepacia* LMG 1222, *B*. *metallica* LMG 24068 and *B*. *cenocepacia* K56-2. Error bars represent SEM. Statistically significant differences are indicated with an asterisk, p < 0.05, n ≥ 3.

Finally, to investigate whether an increase in fluorescence after treatment is strain dependent, other *Bcc* strains were tested (Fig 11). Only for *B*. *metallica* LMG 24068 a significant increase in fluorescence was measured after treatment with Tob. Fluorescence was most increased after treatment with Cip and for *B*. *multivorans* LMG 13010 and *B*. *cepacia* LMG 1222 fluorescence was also significantly higher after treatment with Mer. Overall these results suggest that whether or not fluorescence is higher after treatment is not only lifestyle, time and antibiotic dependent but also strain dependent. This may also explain why no significant difference in fluorescence was observed between Tob treated and untreated cultures for *B*. *cenocepacia* C5424 and the *katB* deletion strain (S3 Fig).The observed differences between strains are in agreement with previous studies and further complicate studying the involvement of ROS in antibiotic mediated killing. Liu et al. (2012) also observed differences among *Staphylococcus aureus* strains [36] and Albesa et al. (2004) found that the ROS production was only higher after treatment with Cip in *Staphylococcus aureus*, *E*. *coli*, and *Enterococcus faecalis* strains sensitive to it [37]. However, for the *Bcc* strains tested we could not find a correlation between the MIC and the production of ROS. Dridi et al. (2015) found that whether or not resistance led to a decreased ROS production in clinical isolates of *Streptococcus pneumoniae* differed between laboratory-derived and naturally-selected antibiotic resistant mutants, suggesting the antibiotic induced production of ROS is not universal and varies according to the genetic background of the strains [38].

Using an *oxyR*:*lux* promoter fusion we found that OxyR was induced both in biofilms (Fig 3) and planktonic cultures (S3 Fig) of *B*. *cenocepacia* K56-2 after treatment with Cip or Mer. The observed differences were again concentration and time dependent, but for Cip and Mer in a concentration of 4 times the MIC the increase in luminescence is similar to the one obtained after treatment with 0.03% H_2_O_2_.

The production of ROS upon treating planktonic cells with Tob or Cip was also confirmed using EPR. After treatment with Tob (4 x MIC) or Cip (4 x MIC) for 30 or 180 min, respectively the amplitude was significantly higher compared to in untreated cells (Fig 5).

Finally, addition of different antioxidants led to a decrease in fluorescence and an increase in survival (Fig 6). However, there were differences between the different antioxidants and antibiotics. While after addition of glutathione or cysteine, survival was significantly higher both in biofilms and planktonic cultures after treatment with Tob or Cip, for mannitol differences were significant only after treating biofilms with Tob. For PDTC survival was significantly higher in combination with Cip but lower in combination with Tob, whereas for sodium pyruvate in biofilms survival was higher in combination with Tob but lower in combination with Cip. In the planktonic cultures differences were not statistically significant (p > 0.05). Questioning the use of antioxidants to evaluate the involvement of ROS in antibiotic mediated killing, Imlay (2015) already pointed to the absence of glutathione peroxidase in *E*. *coli* [7]. Glutathione peroxidase catalyses the reaction between glutathione and H_2_O_2_ or other peroxides and may be necessary to degrade H_2_O_2_ or O^2-^. The genome of *B*. *cenocepacia* K56-2 contains a glutathione peroxidase encoding gene [39] and in *B*. *cenocepacia* J2315 (a strain closely related to *B*. *cenocepacia* K56-2) this peroxidase encoding gene was found to be significantly upregultated (1.6x) after treatment with Tob [19], suggesting involvement in the protection against Tob.

If ROS is involved in antibiotic mediated killing, mutants lacking protection against oxidative stress are expected to be more sensitive to antibiotics, whereas survival would be increased in mutants better protected against oxidative stress. In a previous study we already found that in biofilms formed by a *B*. *cenocepacia* C5424 catalase deletion mutant (Δ*katB*) almost 40 times less cells survived treatment with Tob [19]. Similarly in the present study we found survival after treatment with Cip to be almost 30 times reduced in biofilms formed by the Δ*katB* mutant (S9 Fig). After treatment with Mer, which only has a minor effect on *B*. *cenocepacia*, there were no differences in survival between the WT and the catalase deletion mutant (S9 Fig), suggesting a correlation between ROS production and antibiotic sensitivity.

## Conclusion

Our results highlight some methodological key issues to be considered when evaluating the contribution of ROS in antibiotic mediated killing. Since not a single ideal method could be identified, we recommend to evaluate different methods. Whether or not increased ROS production is measured, is highly dependent on the antibiotics used and the species tested, and even varies between biofilms and planktonic cultures. Nevertheless overall our results suggest that ROS contribute to antibiotic mediated killing in *Bcc* species and that enhancing ROS production or interfering with the protection against ROS may form a novel strategy to improve antibiotic treatment. However, more sensitive and specific methods are needed to obtain a complete understanding of the exact role of ROS in antibiotic mediated killing and to investigate whether the production of ROS is biologically relevant.

## Supporting Information

S1 Fig % surviving cells in *Burkholderia cenocepacia* K56-2 biofilms and planktonic cultures treated with Tob (4 x MIC) (red), Cip (4 x MIC) (orange) or Mer (4 x MIC) (green) for 30 min, 2 h, 6 h or 24 h. Error bars represent SEM. Statistically significant differences compared to untreated are indicated with an asterisk, p < 0.05, n ≥ 3.
(TIF)

S2 Fig H2DCFDA fluorescence in treated versus untreated biofilm and planktonic cultures of *B*. *cenocepacia* K56-2. Cultures were treated with different concentrations of H_2_O_2_ ranging from 0.03 to 3%. Error bars represent SEM. Statistically significant differences are indicated with an asterisk, p < 0.05, n ≥ 3.
(TIF)

S3 Fig H2DCFDA fluorescence after treatment with Tob (4 x MIC, 24 h) or a pH matched control solution. (A) in a *B*. *cenocepacia* C5424 WT and catalase deletion mutant (Δ*katB*) planktonic culture. (B) in a *B*. *cenocepacia* LMG16656 WT and triple quorum sensing deletion mutant planktonic culture. Error bars represent SEM. Statistically significant differences compared to the WT are indicated with an asterisk, p < 0.05, n ≥ 3.
(TIF)

S4 Fig Luminescence generated over time after treating a *B*. *cenocepacia* K56-2 oxyR:lux promoter fusion mutant planktonic culture with 0.03% H2O2, Cip (4 x MIC), Mer (4 x MIC) or Tob (2 x MIC) compared to luminescence in biofilms exposed to LB alone (blue). Data are shown of a single representative experiment.
(TIF)

S5 Fig CTB fluorescence in *Burkholderia cenocepacia* K56-2 biofilms after incubation with different antioxidants. Error bars represent SEM. Statistically significant differences are indicated with an asterisk, p < 0.05, n ≥ 3.
(TIF)

S6 Fig Luminescence generated over time after treating a *B*. *cenocepacia* K56-2 oxyR:lux promoter fusion mutant biofilm (A) and planktonic (B) cultures with Cip (4 x MIC) (orange) or LB alone (blue) compared to luminescence in cultures exposed to Cip (4 x MIC) in combination with an antioxidant. Data are shown of a single representative experiment.
(TIF)

S7 Fig Luminescence generated over time after treating a *B*. *cenocepacia* K56-2 oxyR:lux promoter fusion mutant biofilm culture with 0.03% H_2_O_2_ (blue), 0.03% H_2_O_2_ in combination with PDTC (green) or 0.03% H_2_O_2_ in combination mannitol (red). Data are shown of a single representative experiment.
(TIF)

S8 Fig Fold change in protein carbonylation between treated and untreated planktonic cultures of *B*. *cenocepacia* K56-2. Error bars represent SEM. Statistically significant differences are indicated with an asterisk, p < 0.05, n ≥ 3.
(TIF)

S9 Fig % surviving cells in a *B*. *cenocepacia* C5424 WT and catalase deletion mutant (Δ*katB*) planktonic culture after 24 h of treatment with with Cip (4 x MIC) or Mer (4 x MIC). Error bars represent SEM. Statistically significant differences compared to the WT are indicated with an asterisk, p < 0.05, n ≥ 3.
(TIF)

S1 Table Antioxidants: Mic and concentrations used. (TIF)

## References

[1] Imlay JA, Fridovich I. Superoxide production by respiring membranes of *Escherichia coli*. Free radic Res Commun. 1991;12–13 Pt 1:59–66. 1649104 10.3109/10715769109145768

[2] Wang X, Zhao X. Contribution of oxidative damage to antimicrobial lethality. Antimicrob Agents Chemother. 2009;53(4):1395–402. 10.1128/AAC.01087-08 19223646 PMC2663088

[3] Kohanski MA, Dwyer DJ, Hayete B, Lawrence CA, Collins JJ. A common mechanism of cellular death induced by bactericidal antibiotics. Cell. 2007;130(5):797–810. 17803904 10.1016/j.cell.2007.06.049

[4] Kohanski MA, Dwyer DJ, Wierzbowski J, Cottarel G, Collins JJ. Mistranslation of membrane proteins and two-component system activation trigger antibiotic-mediated cell death. Cell. 2008;135(4):679–90. 10.1016/j.cell.2008.09.038 19013277 PMC2684502

[5] Liu Y, Imlay JA. Cell death from antibiotics without the involvement of reactive oxygen species. Science (New York, NY). 2013;339(6124):1210–3. 10.1126/science.1232751 PMC3731989 23471409

[6] Keren I, Wu Y, Inocencio J, Mulcahy LR, Lewis K. Killing by bactericidal antibiotics does not depend on reactive oxygen species. Science (New York, NY). 2013;339(6124):1213–6. 10.1126/science.1232688 23471410

[7] Imlay JA. Diagnosing oxidative stress in bacteria: not as easy as you might think. Curr Opin Microbiol. 2015;24:124–31. 10.1016/j.mib.2015.01.004 25666086 PMC4380616

[8] Dwyer DJ, Belenky PA, Yang JH, MacDonald IC, Martell JD, Takahashi N, et al Antibiotics induce redox-related physiological alterations as part of their lethality. Proc Natl Acad Sci USA. 2014;111(20):E2100–9. 10.1073/pnas.1401876111 24803433 PMC4034191

[9] Renggli S, Keck W, Jenal U, Ritz D. Role of autofluorescence in flow cytometric analysis of *Escherichia coli* treated with bactericidal antibiotics. J Bacteriol. 2013;195(18):4067–73. 10.1128/JB.00393-13 23836867 PMC3754729

[10] Paez PL, Becerra MC, Albesa I. Antioxidative mechanisms protect resistant strains of *Staphylococcus aureus* against ciprofloxacin oxidative damage. Fundam Clin Pharmacol. 2010;24(6):771–6. 10.1111/j.1472-8206.2009.00806.x 20412315

[11] Chittezham Thomas V, Kinkead LC, Janssen A, Schaeffer CR, Woods KM, Lindgren JK, et al A dysfunctional tricarboxylic acid cycle enhances fitness of *Staphylococcus epidermidis* during beta-lactam stress. mBio. 2013;4(4). 10.1128/mBio.00437-13 PMC3747581 23963176

[12] Kolodkin-Gal I, Sat B, Keshet A, Engelberg-Kulka H. The communication factor EDF and the toxin-antitoxin module mazEF determine the mode of action of antibiotics. PLOS Biol. 2008;6(12):e319 10.1371/journal.pbio.0060319 19090622 PMC2602726

[13] Goswami M, Mangoli SH, Jawali N. Involvement of reactive oxygen species in the action of ciprofloxacin against *Escherichia coli*. Antimicrob Agents Chemother. 2006;50(3):949–54. 16495256 10.1128/AAC.50.3.949-954.2006 PMC1426460

[14] Battan PC, Barnes AI, Albesa I. Resistance to oxidative stress caused by ceftazidime and piperacillin in a biofilm of *Pseudomonas*. Luminescence: the journal of biological and chemical luminescence. 2004;19(5):265–70. 15386799 10.1002/bio.779

[15] Aiassa V, Barnes AI, Albesa I. Resistance to ciprofloxacin by enhancement of antioxidant defenses in biofilm and planktonic *Proteus mirabilis*. Biochem Biophys Res Commun. 2010;393(1):84–8. 10.1016/j.bbrc.2010.01.083 20097163

[16] Wright GD, Hung DT, Helmann JD. How antibiotics kill bacteria: new models needed? Nat Med. 2013;19(5):544–5. 10.1038/nm.3198 23652106

[17] Fang FC. Antibiotic and ROS linkage questioned. Nat Biotech. 2013;31(5):415–6. 10.1038/nbt.2574 23657395

[18] Peeters C, Zlosnik JE, Spilker T, Hird TJ, Lipuma JJ, Vandamme P. *Burkholderia pseudomultivorans* sp. nov., a novel *Burkholderia cepacia* complex species from human respiratory samples and the rhizosphere. Syst Appl Microbiol. 2013;36(7):483–9. 10.1016/j.syapm.2013.06.003 23867250

[19] Van Acker H, Sass A, Bazzini S, De Roy K, Udine C, Messiaen T, et al Biofilm-grown *Burkholderia* cepacia complex cells survive antibiotic treatment by avoiding production of reactive oxygen species. PlOS ONE. 2013;8(3):e58943 10.1371/journal.pone.0058943 23516582 PMC3596321

[20] Cardona ST, Mueller CL, Valvano MA. Identification of essential operons with a rhamnose-inducible promoter in *Burkholderia cenocepacia*. Appl Environ Microbiol. 2006;72(4):2547–55. 16597956 10.1128/AEM.72.4.2547-2555.2006 PMC1448982

[21] El-Halfawy OM, Valvano MA. Putrescine reduces antibiotic-induced oxidative stress as a mechanism of modulation of antibiotic resistance in *Burkholderia cenocepacia*. Antimicrob Agents Chemother. 2014;58(7):4162–71. 10.1128/AAC.02649-14 24820075 PMC4068564

[22] Lefebre MD, Flannagan RS, Valvano MA. A minor catalase/peroxidase from Burkholderia cenocepacia is required for normal aconitase activity. Microbiol (Reading, England). 2005;151(Pt 6):1975–85. 10.1099/mic.0.27704-0 15942004

[23] Udine C, Brackman G, Bazzini S, Buroni S, Van Acker H, Pasca MR, et al Phenotypic and genotypic characterisation of Burkholderia cenocepacia J2315 mutants affected in homoserine lactone and diffusible signal factor-based quorum sensing systems suggests interplay between both types of systems. PlOS ONE. 2013;8(1):e55112 10.1371/journal.pone.0055112 23383071 PMC3557247

[24] EUCAST and ESCMID. Determination of minimum inhibitory concentrations (MICs) of antibacterial agents by agar dilution. Clin Microbiol Infect, 6(9); 2003 p. 509–15. 10.1046/j.1469-0691.2000.00142.x 11168187

[25] Gielis JF, Boulet GA, Briede JJ, Horemans T, Debergh T, Kusse M, et al Longitudinal quantification of radical bursts during pulmonary ischaemia and reperfusiondagger. Eur J Cardiothorac Surg. 2015;48(4):622–9. 10.1093/ejcts/ezu518 25564212

[26] Tavernier S, Coenye T. Quantification of *Pseudomonas aeruginosa* in multispecies biofilms using PMA-qPCR. PeerJ. 2015;3:e787 10.7717/peerj.787 25755923 PMC4349053

[27] Peeters E, Nelis HJ, Coenye T. In vitro activity of ceftazidime, ciprofloxacin, meropenem, minocycline, tobramycin and trimethoprim/sulfamethoxazole against planktonic and sessile *Burkholderia cepacia* complex bacteria. J Antimicrob Chemother. 2009;64(4):801–9. 10.1093/jac/dkp253 19633000

[28] Paulander W, Wang Y, Folkesson A, Charbon G, Lobner-Olesen A, Ingmer H. Bactericidal antibiotics increase hydroxyphenyl fluorescein signal by altering cell morphology. PlOS ONE. 2014;9(3):e92231 10.1371/journal.pone.0092231 24647480 PMC3960231

[29] Jo I, Chung IY, Bae HW, Kim JS, Song S, Cho YH, et al Structural details of the OxyR peroxide-sensing mechanism. Proc Natl Acad Sci USA. 2015;112(20):6443–8. 10.1073/pnas.1424495112 25931525 PMC4443364

[30] Wang Y, Hougaard AB, Paulander W, Skibsted LH, Ingmer H, Andersen ML. Catalase expression is modulated by vancomycin and ciprofloxacin and influences the formation of free radicals in *Staphylococcus aureus* cultures. Appl Environ Microbiol. 2015;81(18):6393–8. 10.1128/AEM.01199-15 26150471 PMC4542236

[31] Dhamdhere G, Krishnamoorthy G, Zgurskaya HI. Interplay between drug efflux and antioxidants in *Escherichia coli* resistance to antibiotics. Antimicrob Agents Chemother. 2010;54(12):5366–8. 10.1128/AAC.00719-10 20876376 PMC2981227

[32] Wang JH, Singh R, Benoit M, Keyhan M, Sylvester M, Hsieh M, et al Sigma S-dependent antioxidant defense protects stationary-phase *Escherichia coli* against the bactericidal antibiotic gentamicin. Antimicrob Agents Chemother. 2014;58(10):5964–75. 10.1128/AAC.03683-14 25070093 PMC4187989

[33] Peeters E, Sass A, Mahenthiralingam E, Nelis H, Coenye T. Transcriptional response of *Burkholderia cenocepacia* J2315 sessile cells to treatments with high doses of hydrogen peroxide and sodium hypochlorite. BMC Genomics. 2010;11. 10.1186/1471-2164-11-90 PMC2830190 20137066

[34] Foti JJ, Devadoss B, Winkler JA, Collins JJ, Walker GC. Oxidation of the guanine nucleotide pool underlies cell death by bactericidal antibiotics. Science (New York, NY). 2012;336(6079):315–9. 10.1126/science.1219192 PMC3357493 22517853

[35] Neeley WL, Essigmann JM. Mechanisms of formation, genotoxicity, and mutation of guanine oxidation products. Chem Res Toxicol. 2006;19(4):491–505. 16608160 10.1021/tx0600043

[36] Liu Y, Liu X, Qu Y, Wang X, Li L, Zhao X. Inhibitors of reactive oxygen species accumulation delay and/or reduce the lethality of several antistaphylococcal agents. Antimicrob Agents Chemother. 2012;56(11):6048–50. 10.1128/AAC.00754-12 22948880 PMC3486621

[37] Albesa I, Becerra MC, Battan PC, Paez PL. Oxidative stress involved in the antibacterial action of different antibiotics. Biochem Biophys Res Commun. 2004;317(2):605–9. 15063800 10.1016/j.bbrc.2004.03.085

[38] Dridi B, Lupien A, Bergeron MG, Leprohon P, Ouellette M. Antibiotic-induced oxidative stress responses between laboratory and clinical isolates of *Streptococcus pneumoniae*. Antimicrob Agents Chemother. 2015. 10.1128/AAC.00316-15 PMC4538540 26100702

[39] Winsor GL, Khaira B, Van Rossum T, Lo R, Whiteside MD, Brinkman FSL. The *Burkholderia* Genome Database: facilitating flexible queries and comparative analyses. Bioinformatics. 2008;24(23):2803–4. 10.1093/bioinformatics/btn524 18842600 PMC2639269

