# Supplementary figures and images for: The Role of Reactive Oxygen Species in Antibiotic-Induced Cell Death in Burkholderia cepacia Complex Bacteria
Source: PLoS One. 2016 Jul 20;11(7):e0159837. doi: 10.1371/journal.pone.0159837 (PMC4954720; doi:10.1371/journal.pone.0159837)

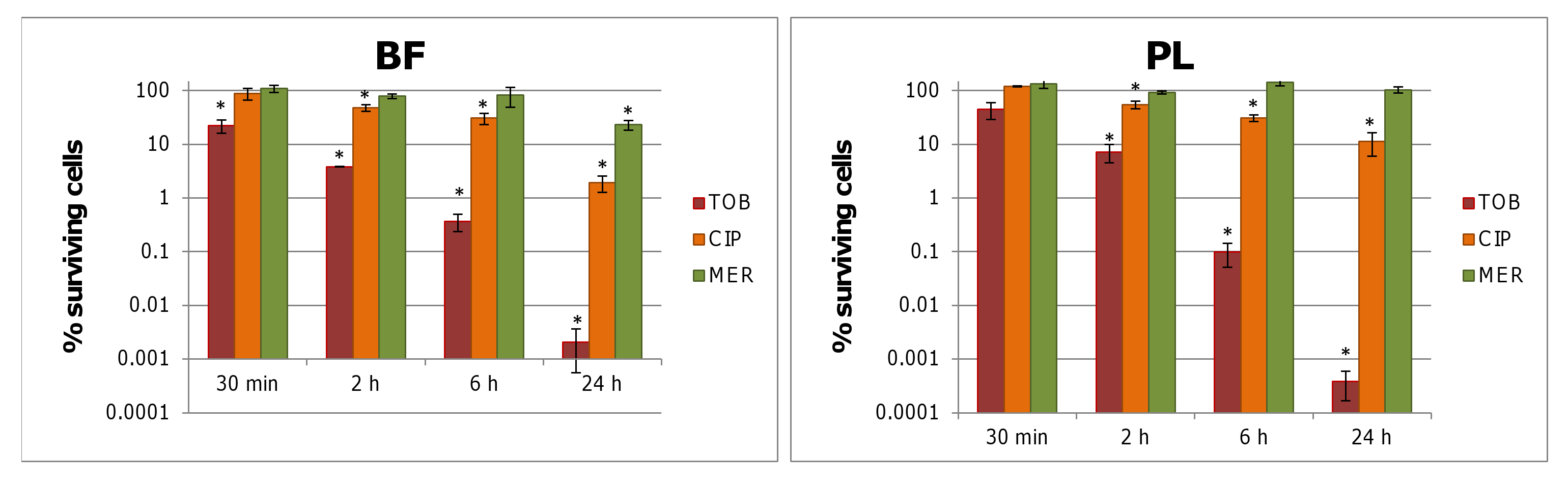

Supplement: S1 Fig — Error bars represent SEM. Statistically significant differences compared to untreated are indicated with an asterisk, p < 0.05, n ≥ 3. (TIF) [file pone.0159837.s001.tif]

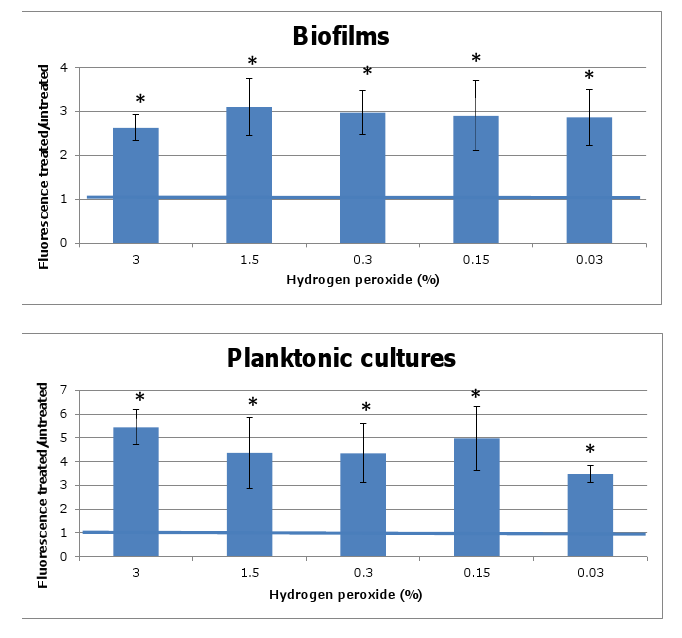

Supplement: S2 Fig — Error bars represent SEM. Statistically significant differences are indicated with an asterisk, p < 0.05, n ≥ 3. (TIF) [file pone.0159837.s002.tif]

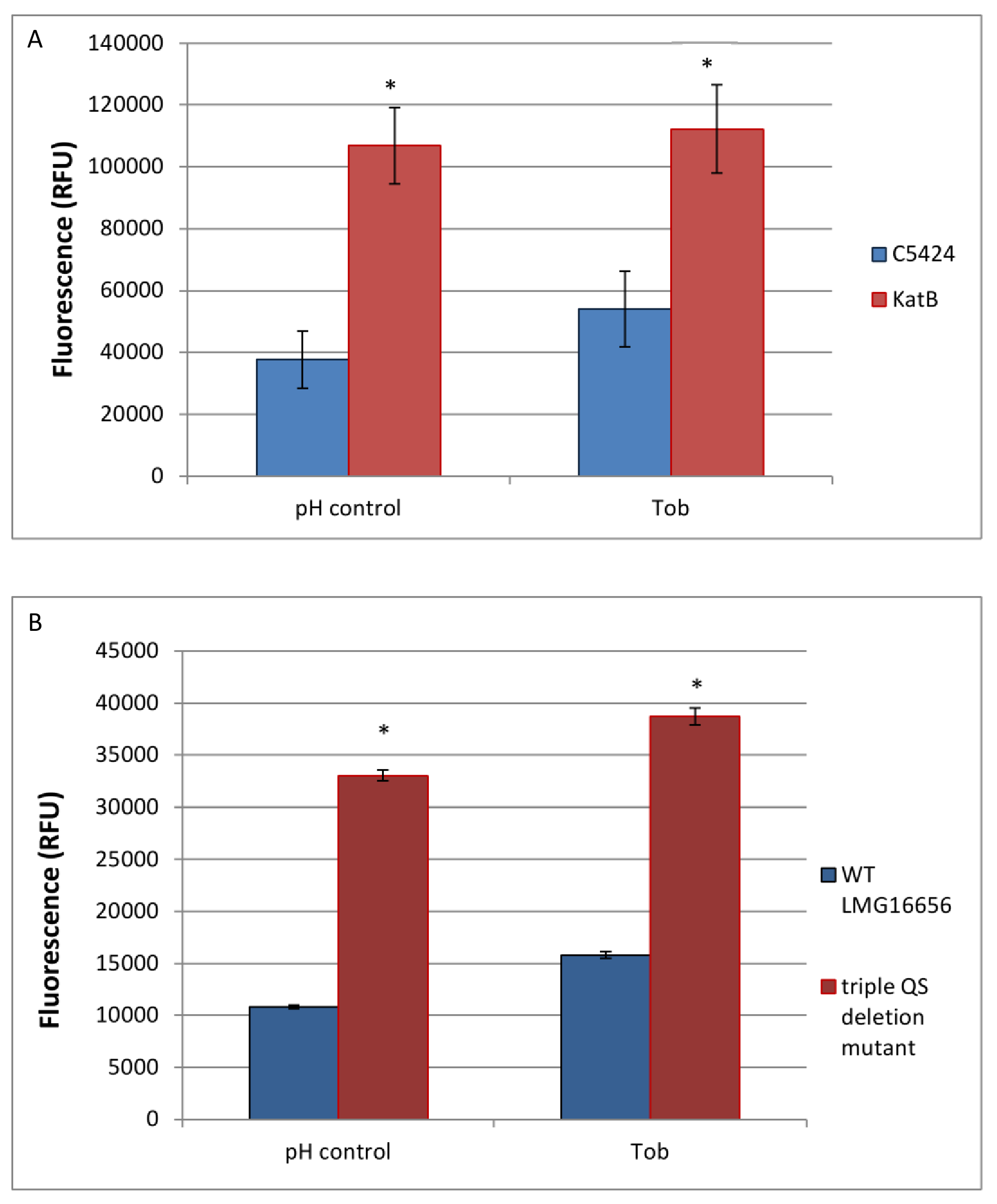

Supplement: S3 Fig — (A) in a B. cenocepacia C5424 WT and catalase deletion mutant (ΔkatB) planktonic culture. (B) in a B. cenocepacia LMG16656 WT and triple quorum sensing deletion mutant planktonic culture. Error bars represent SEM. Statistically significant differences compared to the WT are indicated with an asterisk, p < 0.05, n ≥ 3. (TIF) [file pone.0159837.s003.tif]

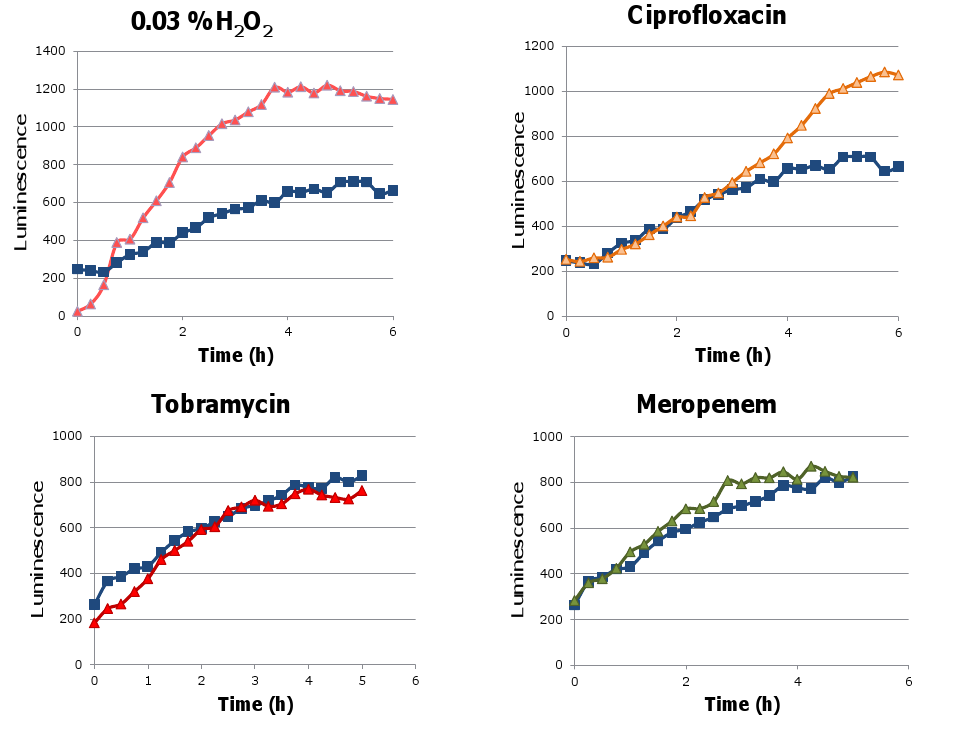

Supplement: S4 Fig — Data are shown of a single representative experiment. (TIF) [file pone.0159837.s004.tif]

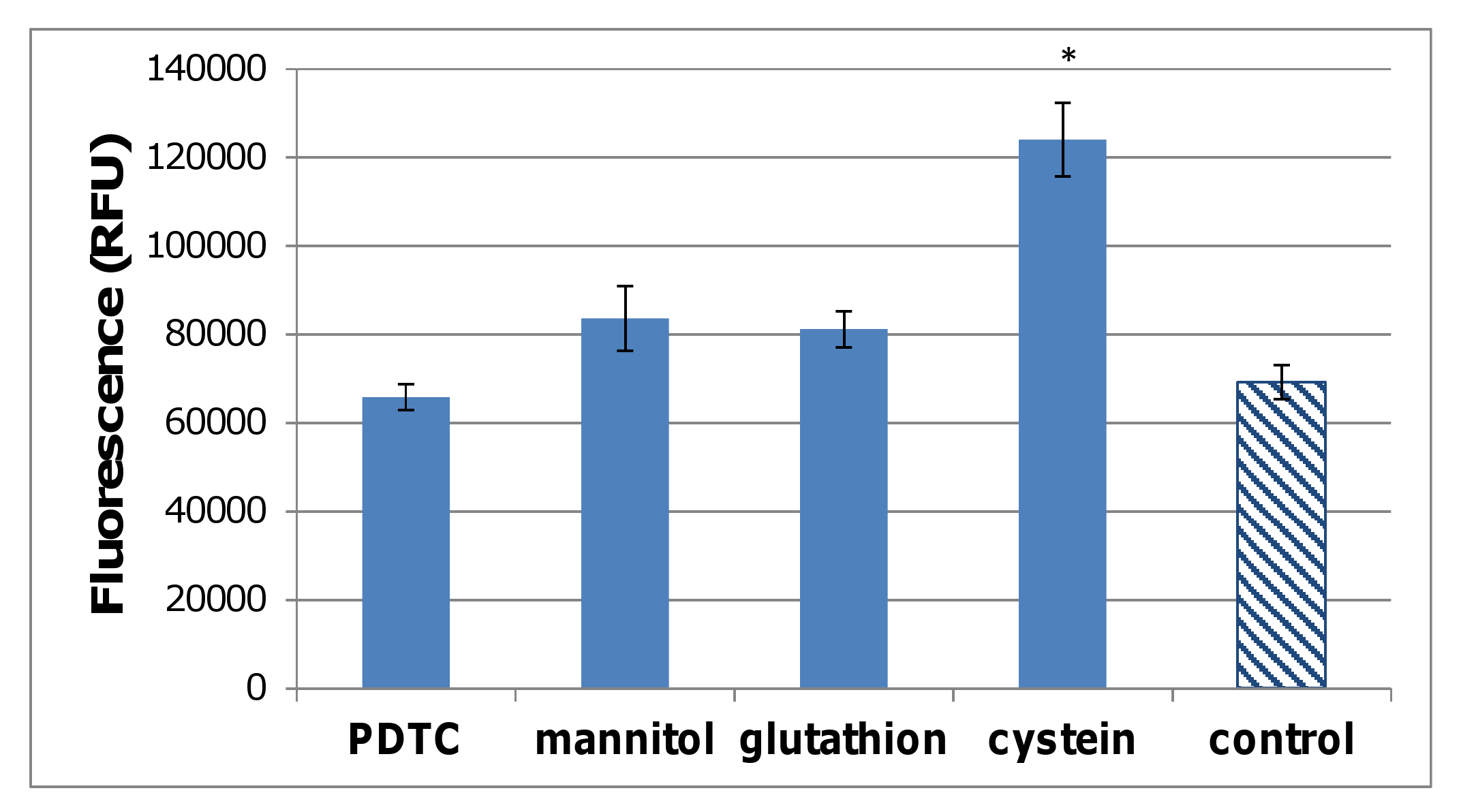

Supplement: S5 Fig — Error bars represent SEM. Statistically significant differences are indicated with an asterisk, p < 0.05, n ≥ 3. (TIF) [file pone.0159837.s005.tif]

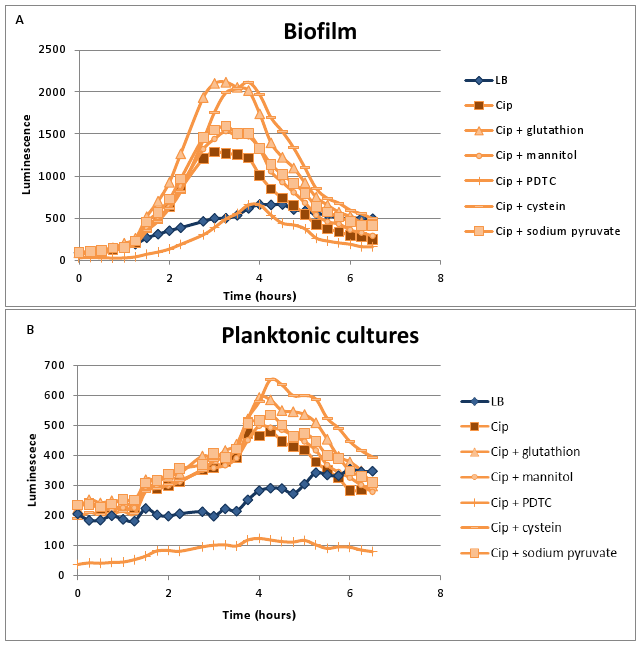

Supplement: S6 Fig — Data are shown of a single representative experiment. (TIF) [file pone.0159837.s006.tif]

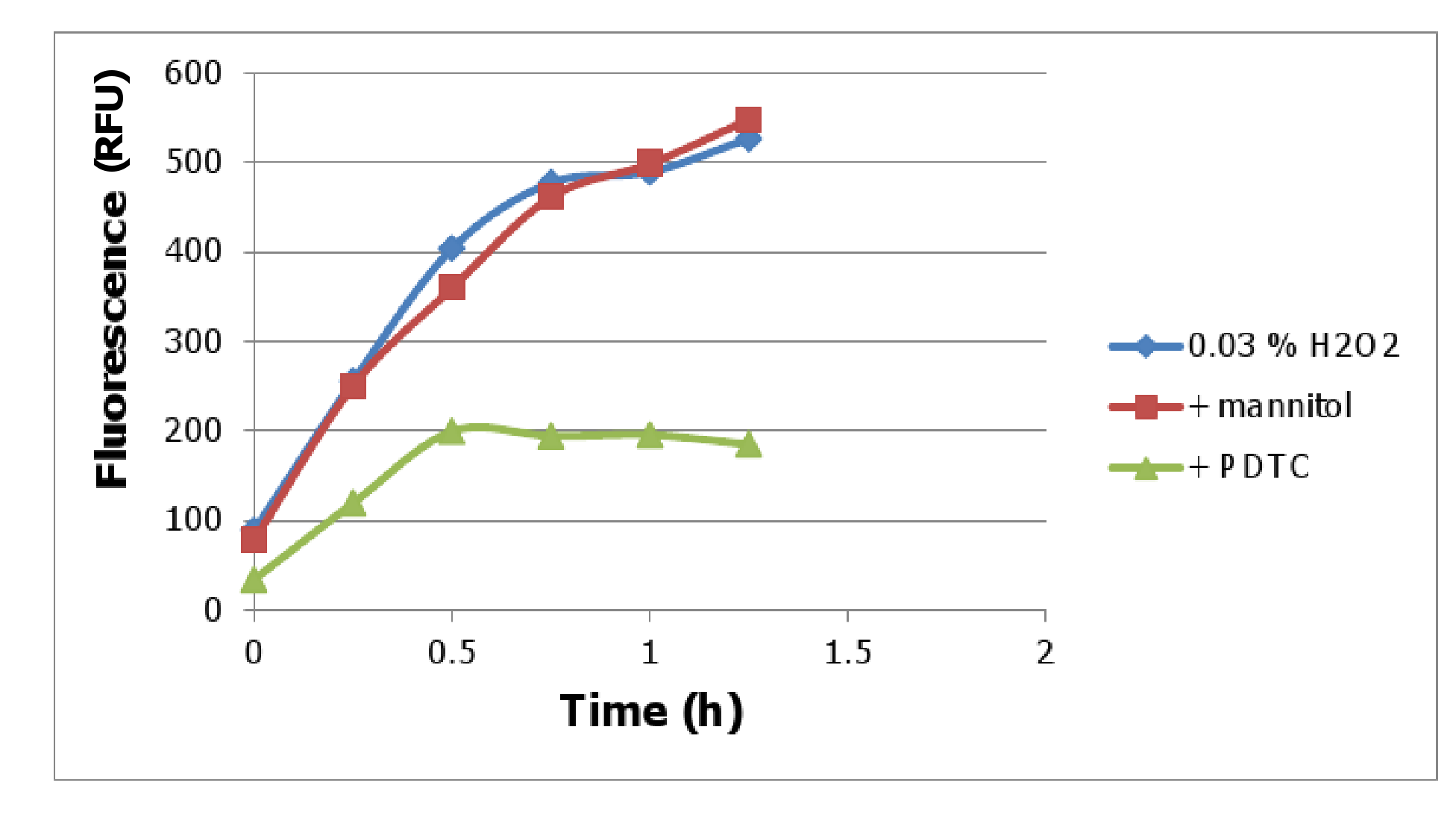

Supplement: S7 Fig — Data are shown of a single representative experiment. (TIF) [file pone.0159837.s007.tif]

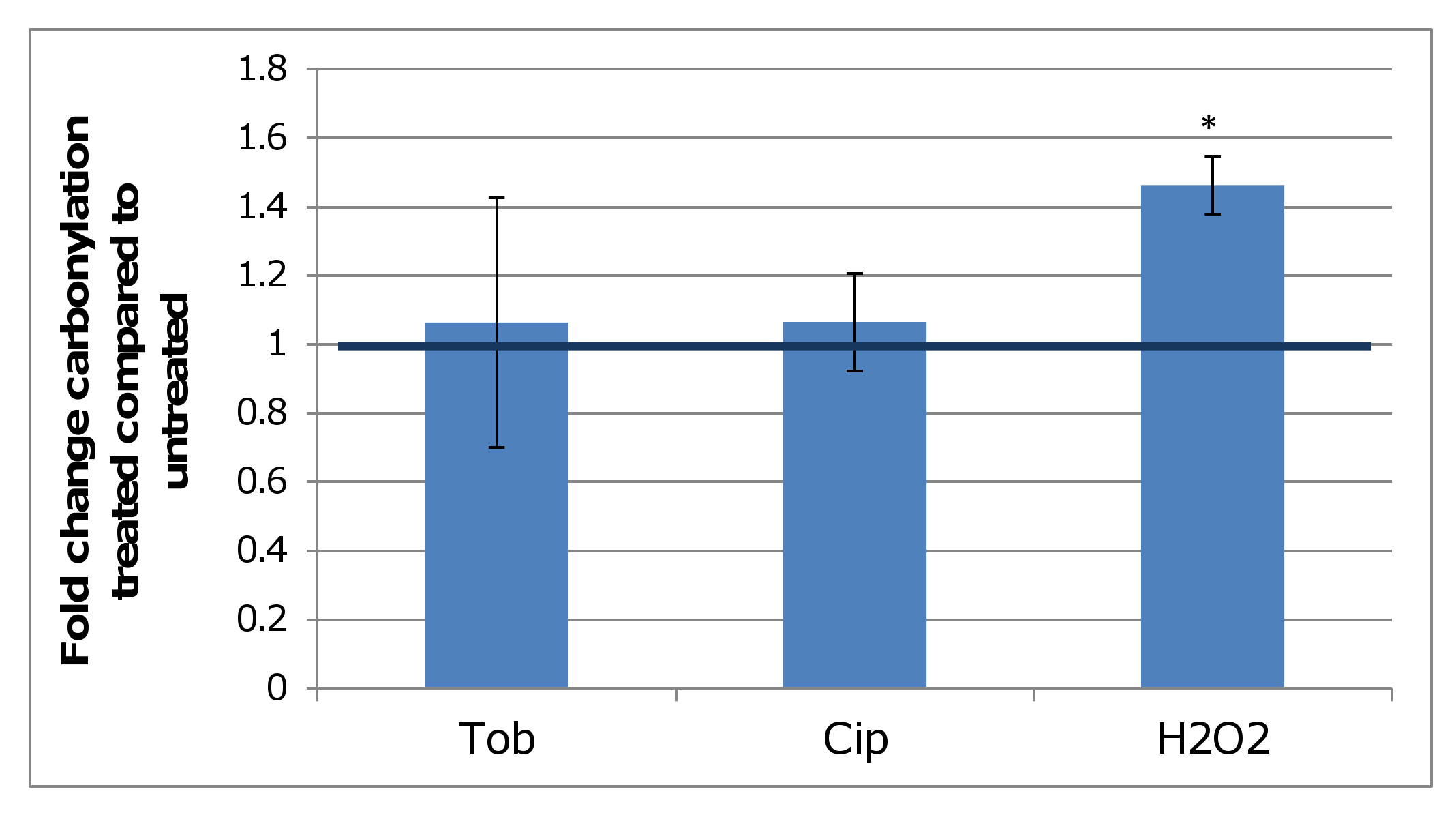

Supplement: S8 Fig — Error bars represent SEM. Statistically significant differences are indicated with an asterisk, p < 0.05, n ≥ 3. (TIF) [file pone.0159837.s008.tif]

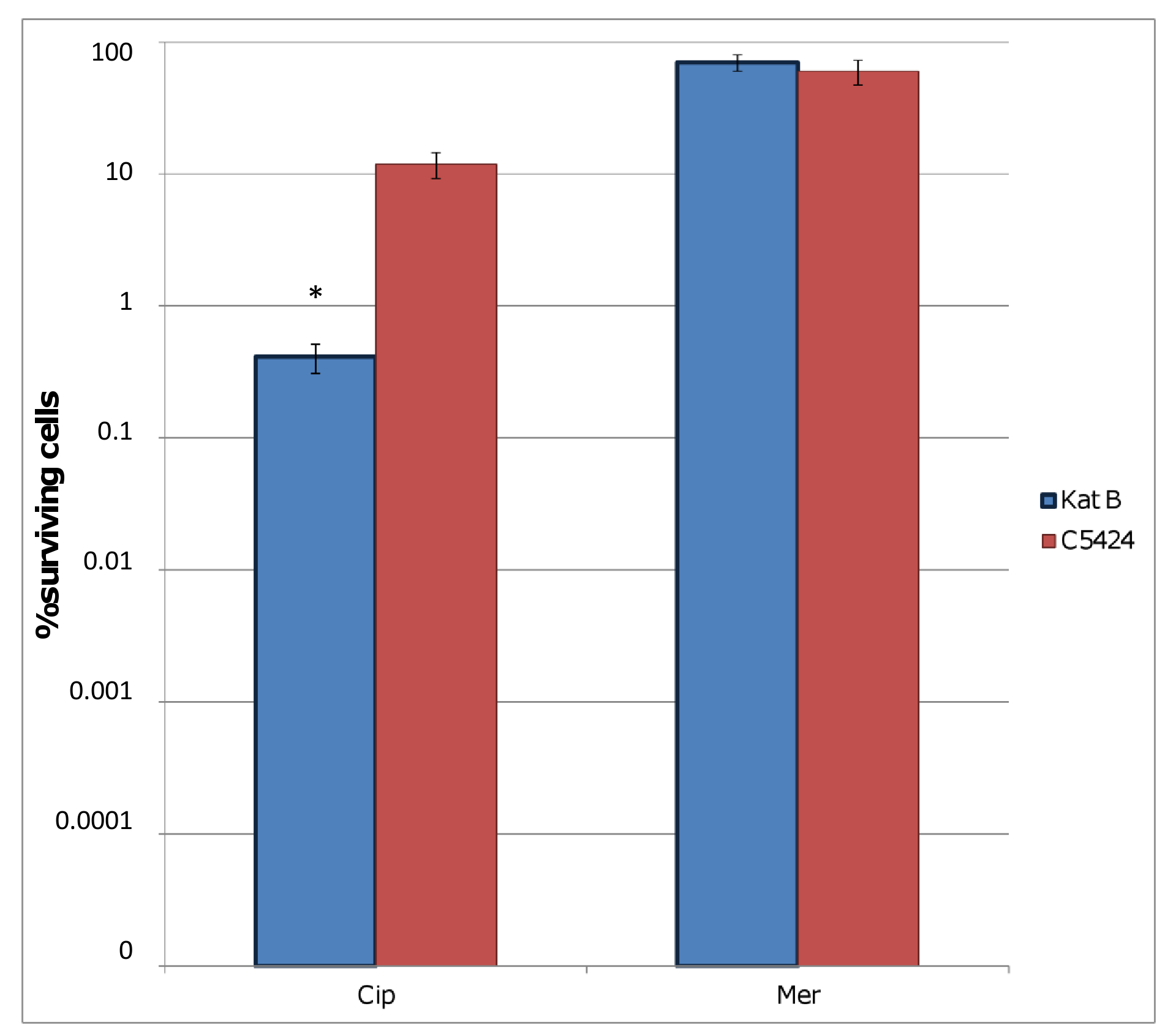

Supplement: S9 Fig — Statistically significant differences compared to the WT are indicated with an asterisk, p < 0.05, n ≥ 3. (TIF) [file pone.0159837.s009.tif]

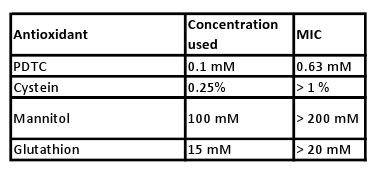

Supplement: S1 Table — (TIF) [file pone.0159837.s010.tif]
